# Exploring the Value of Additional Primary Tumour Excision Combined with Systemic Therapy Administered in Different Sequences for Patients with de Novo Metastatic Breast Cancer

**DOI:** 10.1155/2022/5049445

**Published:** 2022-08-25

**Authors:** Siyi Zhu

**Affiliations:** Department of General Surgery, Shanghai Ninth People's Hospital, Shanghai Jiao Tong University School of Medicine, 639 Zhizaoju Road, Huangpu District, Shanghai 200025, China

## Abstract

**Introduction:**

Uncertainty still remains regarding the survival improvement derived from immediate surgery or subsequent surgery in addition to systemic therapy for patients with de novo metastatic breast cancer. The current study aimed to examine the effect of combined treatment administered in different sequences on the survival of these patients.

**Materials and Methods:**

We conducted a retrospective cohort study of patients with de novo stage IV breast cancer in the Surveillance, Epidemiology, and End Results (SEER) database from 2010 to 2019. Patients were categorized into 3 groups: (1) systemic therapy without primary surgery, (2) systemic therapy after primary surgery, and (3) systemic therapy before primary surgery. Cumulative incidence curves with Gray's test were used to compare breast cancer-specific death (BCSD) between groups. Kaplan–Meier curves with the log-rank test were applied to compare overall survival (OS) between groups. A competing risk model and a proportional hazards model were generated to adjust for important prognostic factors. Propensity score matching (PSM) was performed in the primary survival analysis. Stratified analysis was also performed.

**Results:**

Patients who underwent systemic therapy after primary surgery and who underwent systemic therapy before primary surgery both showed a significantly reduced risk of BCSD compared to patients who received systemic therapy without primary surgery [subdistribution hazard ratio (SHR): 0.74; 95% confidence interval (CI): 0.69–0.79; and *P* < 0.001, and SHR: 0.62; 95% CI: 0.56–0.67; and *P* < 0.001, respectively]. A statistically significant disparity was also noted in OS. In the setting of single-organ metastasis, including the bone, lung, and liver, patients receiving the combination therapy showed an improved prognosis compared with patients receiving systemic therapy without primary surgery.

**Conclusions:**

Additional primary tumour excision, whether before or after systemic therapy, may provide survival benefits for patients presenting with de novo metastatic breast cancer, especially for patients with single-organ disease involving the bone, lung, and liver but not the brain. Further investigations mainly focused on these carefully selected candidates are required to improve personalized treatment for metastatic breast cancer.

## 1. Introduction

Breast cancer is the most common cancer diagnosed in women, and approximately 6% of tumours have metastasized at the first presentation [1, 2]. Systemic therapy is recommended by the NCCN Panel as the primary treatment approach for the management of metastatic breast cancer, while surgery after initial systemic treatment is only considered to treat specific localized problems [3].

Studies have been investigating whether primary tumour excision may provide survival benefits in the setting of de novo metastatic breast cancer over the past two decades. An early retrospective study based on the National Cancer Database (NCDB) first reported a prolonged overall survival (OS) after surgical resection of the primary tumour [4]. Since then, more retrospective studies have claimed a survival advantage associated with primary tumour excision [5, 6]. More importantly, the potential survival benefit provided by primary tumour surgery reportedly increases over time [7]. These findings have challenged the current pattern of performing surgical procedures with palliative intent in the management of de novo stage IV breast cancer. Four randomized controlled trials (RCTs) investigating this treatment modality have attempted to answer the question, but have obtained discrepant results [8–12]. Heterogeneity among the studies and small sample sizes increase the difficulty of reaching a definitive conclusion.

According to the design of prior prospective studies, two studies prescribed subsequent surgery following systemic therapy in the experimental arm [8, 9], while the other two prescribed surgery prior to systemic therapy accordingly [10, 11]. Despite a similar design in ABCSG-28 and MF07-01, conflicting results were obtained regarding the survival improvement of patients receiving upfront surgery [10, 11]. The MF07-01 trial continued to show a survival advantage for patients who underwent upfront surgery at the 10-year follow-up assessment [10]. On the other hand, receipt of upfront surgery, simultaneously resulting in a delay of first-line systemic therapy and inability to monitor drug response, was reported to facilitate cancer metastasis in ABCSG-28 [11]. ESMO guidelines have proposed that only patients with a good response to initial systemic therapy may be offered primary tumour surgery [13]. Therefore, uncertainty still remains regarding the timing and role of primary tumour excision. To date, a limited number of retrospective studies have focused on the effect of the combination of systemic therapy and primary tumour surgery administered in different sequences [6].

The study was thus initiated to examine the effects of upfront surgery and subsequent surgery in addition to systemic therapy compared with systemic therapy without primary surgery on de novo metastatic breast cancer.

## 2. Methods

### 2.1. Data Collection

**Figure 1:**
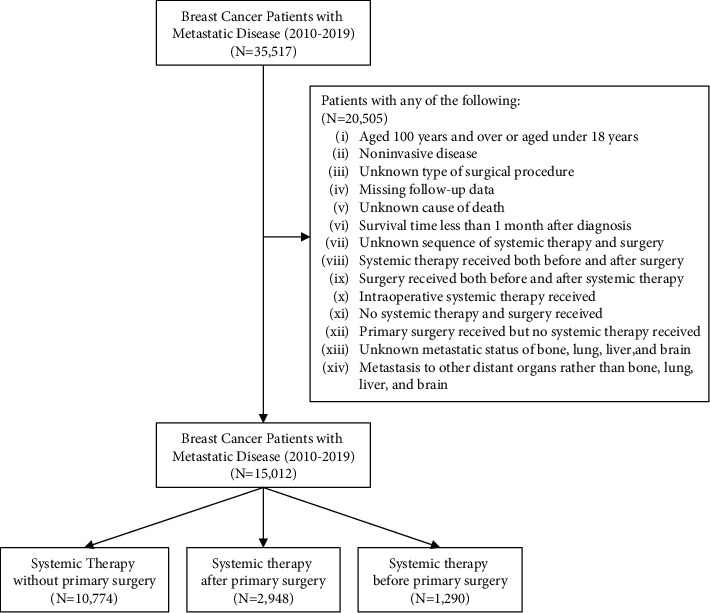
Patient selection flowchart.

Patients with malignant breast tumours from 2010 to 2019 were identified in the Surveillance, Epidemiology, and End Results (SEER) database: *Incidence—SEER Research Plus Data, 17 Registries, Nov 2021 Sub (2000–2019)* using SEER^*∗*^Stat software (version 8.3.9.2) [14]. Patients with metastatic disease were further selected from the patients above (*N* = 35,517). All included patients were aged over 18 years and under 100 years. Patients with noninvasive disease, an unknown sequence of systemic therapy and surgery, an unknown type of surgical procedure, missing follow-up data, unknown cause of death, or survival time less than 1 month after diagnosis were excluded from the analysis. Patients who received systemic therapy both before and after surgery, who received surgery both before and after systemic therapy, who received intraoperative systemic therapy, who received surgery of the primary site without systemic therapy, and who did not receive any systemic therapy or surgery of the primary site were also excluded. Moreover, patients with unknown metastatic status of the bone, brain, liver, and lung and patients who had metastasis to other distant organs rather than the bone, brain, liver, and lung were excluded. Ultimately, patients were categorized into 3 groups: (1) systemic therapy without primary surgery, (2) systemic therapy after primary surgery, and (3) systemic therapy before primary surgery. The flowchart of the patient selection process is presented in Figure 1.

### 2.2. Statistical Analysis

Baseline characteristics were compared across treatment groups using the Pearson's chi-squared test for categorical variables. The percentage of patients receiving each treatment modality was plotted against the year of diagnosis to reflect the overall trend of adopting each modality. A multivariate logistic regression model was generated to evaluate the correlation between treatment sequence and type of surgical procedure among patients undergoing surgery of the primary site. The survival outcomes measured in this study were breast cancer-specific survival (BCSS) and OS. Cumulative incidence curves of breast cancer-specific death (BCSD) were plotted and compared using Gray's test. Kaplan–Meier curves of OS were plotted and compared using the log-rank test. In multivariate analyses of BCSS and OS, Fine–Gray regression models and Cox regression models were implemented to identify the independent prognostic variables. In the primary survival analysis, propensity score matching (PSM) was employed to reduce confounding biases. In the subset analysis, the patients were stratified according to the metastatic site, age, tumour subtype, grade, and disease stage. A two-sided *P*-value <0.05 was considered statistically significant. All statistical analyses were performed using R (version 4.1.1).

## 3. Results

Of the 35,517 patients diagnosed with stage IV breast cancer from 2010 to 2019, 15,012 met the inclusion criteria and were analysed in the study, including 10,774 patients receiving systemic therapy without primary surgery, and the remaining 2,948 and 1,290 patients receiving systemic therapy after and before primary surgery, respectively.

### 3.1. Baseline Characteristics

For the entire study population, the median age was 59 years [interquartile range (IQR) 50−68 years]. A total of 10,644 patients (70.9%) were diagnosed with IDC. The primary tumour was graded as 3 or 4 in 6,110 patients (40.7%). The subtype was reported as HR+/HER2− in 52.4%, HR+/HER2+ in 16.9%, HR−/HER2+ in 9.5%, HR−/HER2− in 13.6%, and unknown in 7.7%. Metastases to the bone, lung, liver, and brain were observed in 74.3%, 34.8%, 30.5%, and 8.4% of the study patients, respectively. The baseline characteristics of the 3 groups are shown in Table 1.

### 3.2. Temporal Trends of Treatment Modalities

**Figure 2:**
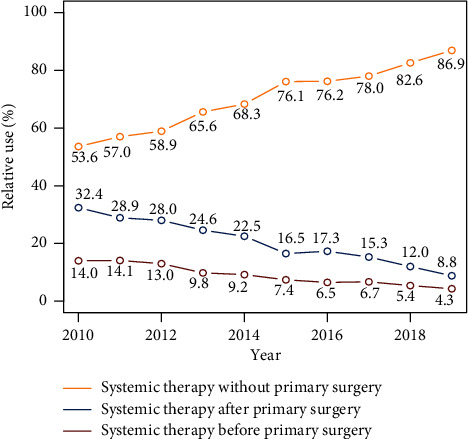
Relative proportion of patients receiving each treatment modality from 2010 to 2019.

Figure 2 illustrates the relative proportion of patients receiving each treatment modality from 2010 to 2019. A significantly increasing trend in the use of systemic therapy only was observed from 53.6% in 2010 to 86.9% in 2019 (*P* for trend <0.001), whereas the trend was downwards for the proportion of patients receiving the other two treatment modalities during the same period.

### 3.3. Treatment Sequence and Surgical Procedure

In the multivariate analysis of patients who had surgery of the primary site, compared to patients receiving systemic therapy after surgery, those receiving systemic therapy first were more likely to undergo radical/extended radical mastectomy [odds ratio (OR): 2.27; 95% CI: 2.22–2.31], modified radical mastectomy (OR: 2.23; 95% CI: 1.90–2.63), or subcutaneous/simple mastectomy (OR: 2.32; 95% CI: 1.97–2.74) (Table 2).

### 3.4. Primary Survival Analysis

**Figure 3:**
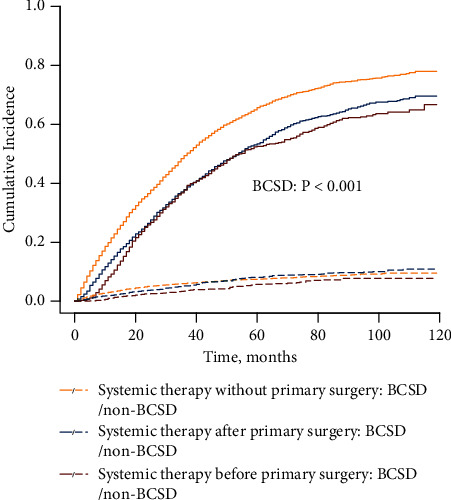
Cumulative incidence curves of BCSD for patients receiving different treatment modalities in the whole study population.

The cumulative incidence curve shows the lowest unadjusted BCSD in patients who underwent combined treatment with systemic therapy and primary tumour surgery (Figure 3). In the adjusted competing risk model, combination therapy remained a favourable prognostic factor compared with systemic therapy only (systemic therapy after primary surgery: SHR: 0.74; 95% CI: 0.69–0.79; and *P* < 0.001, and systemic therapy before primary surgery: SHR: 0.62; 95% CI: 0.56–0.67; and *P* < 0.001) (Table 3). Moreover, patients receiving systemic therapy before primary surgery had a significantly reduced risk of BCSD than those undergoing upfront surgery followed by systemic therapy (SHR: 0.83; 95% CI: 0.76–0.62; and *P* < 0.001).

**Figure 4:**
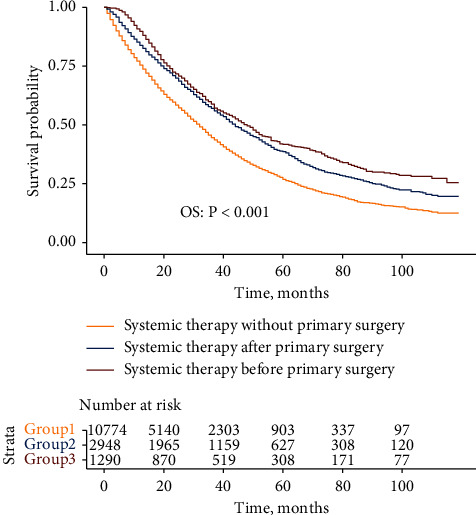
Kaplan–Meier curves of overall survival for patients receiving different treatment modalities in the whole study population.

The Kaplan–Meier curve revealed the greatest improvement in OS for patients who underwent systemic therapy before primary surgery, followed by patients who underwent systemic therapy after primary surgery and those who underwent systemic therapy without primary surgery in sequence (5-year OS: 41.8%, 38.6%, and 26.9%, respectively) (Figure 4). The results were confirmed by the multivariate analysis using a Cox proportional hazards model (Supplementary Table 1).

The multivariate analysis of BCSS and OS in the whole study population also revealed that patients who received non-primary surgical procedure to distant site had a significantly reduced risk of BCSD (SHR: 0.80; 95% CI: 0.69–0.93; and *P*=0.003) and all-cause death (HR: 0.76; 95% CI: 0.66–0.87; and *P* < 0.001) compared to those who did not (Table 3 and Supplementary Table 1).

Propensity score matching was performed to exclude the imbalance of baseline characteristics between different treatment groups. After 1 : 1 matching for patient, tumour, metastatic, and treatment characteristics, 2609 and 1283 patients receiving systemic therapy without primary surgery were identified to be compared with patients in the groups receiving systemic therapy after and before surgery, respectively. The distribution of baseline characteristics was well balanced as shown in Supplementary Tables 2 and 3. The results of the survival analysis after PSM were consistent with those obtained before PSM. Compared with patients in the nonsurgery group, patients undergoing systemic therapy after surgery showed a significantly reduced risk of BCSD (SHR: 0.75; 95% CI: 0.69–0.82; and *P* < 0.001) and all-cause death (HR: 0.74; 95% CI: 0.68–0.80; and *P* < 0.001) after PSM. A similar finding was observed for patients undergoing systemic therapy before surgery with respect to BCSD (SHR: 0.55; 95% CI: 0.49–0.62; and *P* < 0.001) and all-cause death (HR: 0.60; 95% CI: 0.53–0.68; and *P* < 0.001) (Supplementary Tables 4 and 5)

### 3.5. Subgroup Analysis

**Figure 5:**
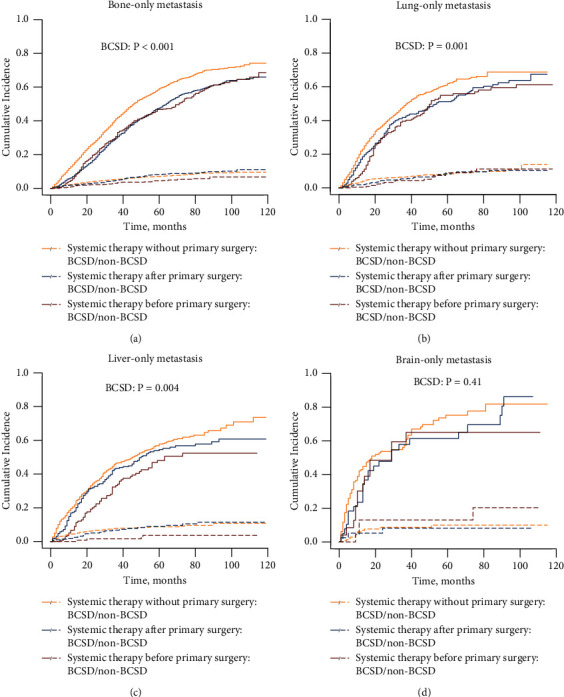
Cumulative incidence curves of BCSD in patients with single-organ disease involving the bone (a), lung (b), liver (c), and brain (d).

The effects of different treatment modalities on survival were further evaluated according to the metastatic site in patients with single-organ involvement (Figure 5 and Supplementary Figure 1). For patients with bone-only involvement, systemic therapy after or before primary surgery provided a statistically significant survival advantage with respect to both BCSS and OS compared to systemic therapy without primary surgery (systemic therapy after surgery: SHR for BCSS: 0.66; 95% CI: 0.60–0.74; HR for OS: 0.68; 95% CI: 0.62–0.75; systemic therapy before surgery: SHR for BCSS: 0.61; 95% CI: 0.53–0.69; HR for OS: 0.56; 95% CI: 0.49–0.64) (Table 4 and Supplementary Table 6). Similar results were obtained for patients with liver-only and lung-only metastases. No difference was observed between combination therapy and monotherapy for patients with brain-only metastases (Figure 5 and Supplementary Figure 1). After comparing the two treatment modalities administered in different sequences, systemic therapy before surgery was superior to systemic therapy after surgery in the whole study population and the subgroup of patients with lung-only metastasis in terms of BCSS.

In terms of the effect of distant site surgery on patients with single-organ metastasis, multivariate analysis in patients with lung-only metastasis showed that receipt of distant site surgery approached the borderline of significance in reducing the risk of BCSD (SHR: 0.61; 95% CI: 0.36–1.03; and *P*=0.07) and reached the level of significance in reducing the risk of all-cause death (HR: 0.47; 95% CI: 0.29–0.75; and *P*=0.002). For patients with single-organ metastasis involving the bone, liver, or brain, the impact of distant site surgery on survival was not statistically significant.

In the subgroup of patients younger than 40 years old, a numerical trend was observed in favour of systemic therapy after primary surgery compared with systemic therapy alone in terms of BCSS (SHR: 0.81; 95% CI: 0.64–1.03; and *P*=0.09). Except for this young subgroup, in any subgroup based on age, tumour subtype, grade, and disease stage, the addition of primary tumour excision was associated with significantly prolonged BCSS and OS, regardless of the sequence of primary surgery and systemic therapy (Supplementary Figures 2 and 3). In the setting of multiple-organ involvement, combined treatment brought both significant BCSS and OS advantages to patients with bone plus liver involvement compared to monotherapy.

## 4. Discussion

We performed a retrospective population-based cohort study to assess the value of primary tumour excision before and after systemic therapy in patients with de novo stage IV breast cancer. Our results revealed a statistically significant association between primary tumour excision before or after systemic therapy and prolonged BCSS and OS. A subgroup analysis based on the metastatic site found that patients with single-organ-involved diseases, including bone, lung, and liver metastasis, may derive a survival benefit. In any subgroup based on age, tumour subtype, tumour differentiation, and disease stage, a combination of systemic therapy and primary tumour surgery resulted in superior survival compared to systemic therapy alone in terms of both BCSS and OS.

In the pooled analysis of three related RCTs comprising 880 patients, their overall survival did not differ by the addition of surgical treatment in the primary breast site [15]. Pooling reported data on locoregional progression and distant progression showed that locoregional treatment exerted a favourable effect on time to locoregional progression and an unfavourable effect on time to distant progression [9, 11, 15]. No prospective studies have reported results on BCSS, while our study lacks data on locoregional or distant progression, and thus a comparison is unrealistic.

As the first RCT published on this topic, the results derived from the Indian population suggested a similar survival benefit derived from surgery after systemic therapy and nonsurgery treatment. The patients were believed to be diagnosed late and undertreated because most of the patients had symptomatic disease, and 92% of HER2+ patients did not receive HER2-targeted therapy. The 2-year survival of the nonsurgery group in their study was also absolutely lower than that in our study (43.0% vs. 58.4%) [9]. The latest update of data from the MF07-01 trial showed prolonged survival with additional upfront surgery after 10 years of follow-up, consistent with the results obtained after a median follow-up period of 40 months. In contrast, no difference was noted in 36-month survival. The weakness of the MF07-01 trial was poor randomization by biomarker status, specifically referring to more patients with HR + tumours and fewer patients with triple-negative disease in the surgery group [10, 12]. The ABCSG-28 trial with a similar study design to MF07-01 yielded no survival benefits with primary tumour surgery, and its insufficient recruitment made it underpowered to provide firm conclusions [11]. The most recently published trial reported that locoregional therapy had no effect on overall survival but had a favourable effect on locoregional control [8].

Although a substantial number of observational studies have confirmed that primary tumour excision might provide additional survival benefits, they had different opinions on potential beneficiaries of the surgical intervention [16–19]. Other matched-pair analyses did not identify any survival difference between the surgery group and the nonsurgery group and thus attributed the survival advantage of primary tumour excision to case selection bias [20, 21]. A prior study accounting for the treatment sequence of locoregional surgery and systemic therapy reported a noticeable survival improvement with primary tumour excision, regardless of whether surgery was performed before or after systemic therapy, but the authors did not compare the survival outcomes of these two groups or perform any subgroup analyses [6]. Another study also confirmed the advantageous effect of primary tumour resection on survival and found no survival difference between the two treatment groups according to the timing of surgery [22]. The current study suggested that systemic therapy before surgery was superior to systemic therapy after surgery in the whole study population and the subgroup of patients with lung-only metastasis, which deserves further research.

Patients undergoing systemic therapy before surgery may represent a subpopulation with a favourable prognosis since they putatively respond well to systemic therapy. Moreover, in consideration of the foregoing limitations of upfront surgery, patients receiving systemic therapy before surgery were distinguished from patients undergoing upfront surgery. In two prospective trials where patients received immediate systemic therapy before randomization, only patients without disease progression were admitted to subsequent trials [8, 9]. In the current study, patients with rapid progression after initial systemic therapy were most likely to be included in the group receiving systemic therapy alone, which may exaggerate the effect of systemic therapy before surgery compared with nonsurgery on survival. On the other hand, patients undergoing upfront surgery also included patients who responded poorly to systemic therapy. Hence, the results suggesting that systemic therapy plus primary tumour excision is superior to systemic therapy alone are convincing to some extent.

Regarding the metastatic pattern, the current study focused on a more limited disease, which made it easier to interpret the potential benefits of surgery. Moreover, patients with metastasis to a single distant organ accounted for over half of the study population. In the initial analysis before adopting the present stricter exclusion criteria, distant lymph node metastasis (DLNM) was shown to be unrelated to the survival outcomes of patients with de novo stage IV breast cancer. A prior SEER-based study also concluded that patients with de novo stage IV breast cancer along with DLNM only had similar survival to patients with N3C disease [23]. Therefore, the status of DLNM was not considered. Here, better survival outcomes were obtained from a combination of primary tumour surgery and systemic therapy in patients with single distant organ metastasis including the bone, liver, and lung, but not the brain, which was similar to another SEER-based analysis [17]. The MF07-01 trial indicated the protective effect of initial surgery on the subgroup with solitary bone-only metastasis but not on the subgroup with multiple bone-only metastases [12]. Because the number of lesions in a single organ was not available, the effect of the metastatic burden on the potential effectiveness of surgical procedures in patients with single-organ metastasis was unable to be determined.

Pooling data for the subgroup analysis of patients stratified by tumour subtype from prospective trials showed that primary tumour surgery did not provide a survival benefit for patients with any tumour subtype, which was the exact opposite of the results in the current study [15]. The general availability of HER2-directed therapy may decrease the magnitude of the effect on locoregional progression [15]. However, the claim was not substantiated here since patients with HER2+ disease experienced longer survival than patients diagnosed with luminal A (HER2−) disease, which indicated the general availability of HER2-directed therapy. Moreover, retrospective studies have documented prolonged survival with surgical interventions for the primary breast tumour site in the presence of effective HER2-directed therapy [24–27]. Tremendous advances in systemic treatment may also play a large role in the beneficial effect of primary tumour surgery on triple-negative disease. A subgroup analysis based on tumour differentiation proved increased BCSS and OS with surgical treatment in all subgroups, consistent with the results based on the tumour subtype.

The effect of distant site surgery on survival was also evaluated in patients with single-organ involvement. Results suggested that patients with lung-only metastasis had the potential to benefit from local therapy of the metastatic lesions to the lung. But the proportion of patients undergoing distant site surgery was only 3.2% in the subgroup of lung-only involvement, which weakened the credibility of the results.

The concept of breast cancer stem cells (CSCs) may partially explain why primary tumour excision improves the oncologic outcomes of patients presenting with de novo metastatic breast cancer. Breast CSCs, constituting up to 35% of the cancer cells in a tumour [28], are considered the root of tumour relapse and drug resistance [29, 30]. Primary tumour excision rapidly eliminates breast CSCs. The assumption is strengthened by early evidence suggesting that the survival of women who received surgery with positive margins was comparable to that of women who received nonsurgical treatment [19]. Other underlying mechanisms include restoration of suppressed immunocompetence [31] and a decrease in the overall tumour burden [32].

This study represents the only analysis comparing the effect of combination therapy administered in different sequences with monotherapy on de novo metastatic breast cancer using the SEER database. Patients were collected in the recent period when more effective novel systemic therapies were available, which may be supported by the growing trend of prescribing systemic therapy alone, as shown in Figure 2. However, limitations still exist. First, selection bias exists in this study due to its retrospective nature. Adjustments for main prognostic factors and PSM were applied to avoid the imbalance of baseline characteristics. Second, treatment details, such as the type of systemic therapy and response to medication, were not considered due to the lack of this information in the SEER database. Third, the mean duration of follow-up was 28.6 months, which is much shorter than previous prospective studies [8–11].

## 5. Conclusions

In conclusion, the current study suggests that receipt of additional primary tumour excision before or after systemic therapy may provide survival benefits for patients presenting with de novo metastatic breast cancer, especially for those with single-organ involvement, including the bone, lung, and liver. Additional randomized clinical trials and high-quality observational studies are required to evaluate the value of primary tumour excision for these specific candidates.

## Figures and Tables

**Table 1:** Demographic, clinicopathologic, and treatment characteristics of the included patients.

Variables	All patients (*N* = 15012)	Systemic therapy without primary surgery (*N* = 10774)	Systemic therapy after primary surgery (*N* = 2948)	Systemic therapy before primary surgery (*N* = 1290)
Age, y				
(18, 40)	1271 (8.5)	907 (8.4)	221 (7.5)	143 (11.1)
(40, 60)	6244 (41.6)	4511 (41.9)	1079 (36.6)	654 (50.7)
(60, 100)	7497 (49.9)	5356 (49.7)	1648 (55.9)	493 (38.2)
Median (IQR)	59 (50–68)	59 (50–68)	62 (51–71)	56 (47–64)
Marital status				
Unmarried	7199 (48.0)	5244 (48.7)	1359 (46.1)	596 (46.2)
Married	7125 (47.5)	5058 (46.9)	1445 (49.0)	622 (48.2)
Unknown	688 (4.6)	472 (4.4)	144 (4.9)	72 (5.6)
Race				
White	11197 (74.6)	7938 (73.7)	2316 (78.6)	943 (73.1)
Black	2415 (16.1)	1794 (16.7)	397 (13.5)	224 (17.4)
Other	1333 (8.9)	985 (9.1)	228 (7.7)	120 (9.3)
Unknown	67 (0.4)	57 (0.5)	7 (0.2)	3 (0.2)
Sex				
Female	14831 (98.8)	10666 (9.0)	2889 (98.0)	1276 (98.9)
Male	181 (1.2)	108 (1.0)	59 (2.0)	14 (1.1)
Histologic type				
IDC	10644 (70.9)	7494 (69.6)	2125 (72.1)	1025 (79.5)
ILC	1499 (10.0)	1042 (9.7)	368 (12.5)	89 (6.9)
Other	2869 (19.1)	2238 (20.8)	455 (15.4)	176 (13.6)
Grade				
I	937 (6.2)	636 (5.9)	214 (7.3)	87 (6.7)
II	5094 (33.9)	3582 (33.2)	1095 (37.1)	417 (32.3)
III/IV	6110 (40.7)	4053 (37.6)	1398 (47.4)	659 (51.1)
Unknown	2871 (19.1)	2503 (23.2)	241 (8.2)	127 (9.8)
AJCC *T* category				
0	240 (1.6)	237 (2.2)	2 (0.1)	1 (0.1)
1	1802 (12.0)	1206 (11.2)	480 (16.3)	116 (9.0)
2	4390 (29.2)	2776 (25.8)	1257 (42.6)	357 (27.7)
3	2305 (15.4)	1534 (14.2)	532 (18.0)	239 (18.5)
4	4405 (29.3)	3319 (30.8)	576 (19.5)	510 (39.5)
X	1870 (12.5)	1702 (15.8)	101 (3.4)	67 (5.2)
AJCC *N* category				
0	3199 (21.3)	2369 (22.0)	628 (21.3)	202 (15.7)
1	6764 (45.1)	5242 (48.7)	925 (31.4)	597 (46.3)
2	1649 (11.0)	816 (7.6)	620 (21.0)	213 (16.5)
3	2278 (15.2)	1378 (12.8)	665 (22.6)	235 (18.2)
X	1122 (7.5)	969 (9.0)	110 (3.7)	43 (3.3)
Molecular subtype				
HR+/HER2−	7862 (52.4)	5428 (50.4)	1828 (62.0)	606 (47.0)
HR+/HER2+	2534 (16.9)	1908 (17.7)	412 (14.0)	214 (16.6)
HR−/HER2+	1422 (9.5)	1067 (9.9)	214 (7.3)	141 (10.9)
HR−/HER2−	2037 (13.6)	1448 (13.4)	334 (11.3)	255 (19.8)
Unknown	1157 (7.7)	923 (8.6)	160 (5.4)	74 (5.7)
Bone involvement				
No	3856 (25.7)	2599 (24.1)	812 (27.5)	445 (34.5)
Yes	11156 (74.3)	8175 (75.9)	2136 (72.5)	845 (65.5)
Lung involvement				
No	9791 (65.2)	6796 (63.1)	2125 (72.1)	870 (67.4)
Yes	5221 (34.8)	3978 (36.9)	823 (27.9)	420 (32.6)
Liver involvement				
No	10438 (69.5)	7160 (66.5)	2270 (77.0)	1008 (78.1)
Yes	4574 (30.5)	3614 (33.5)	678 (23.0)	282 (21.9)
Brain involvement				
No	13746 (91.6)	9696 (90.0)	2814 (95.5)	1236 (95.8)
Yes	1266 (8.4)	1078 (10.0)	134 (4.5)	54 (4.2)
Site of metastasis				
Bone only	6214 (41.4)	4056 (37.6)	1537 (52.1)	621 (48.1)
Viscera only	3415 (22.7)	2249 (20.9)	755 (25.6)	411 (31.9)
Bone + viscera	4117 (27.4)	3391 (31.5)	522 (17.7)	204 (15.8)
Brain involvement	1266 (8.4)	1078 (10.0)	134 (4.5)	54 (4.2)
Number of sites of metastasis				
1	9447 (62.9)	6153 (57.1)	2264 (76.8)	1030 (79.8)
2	4121 (27.5)	3355 (31.1)	555 (18.8)	211 (16.4)
≥3	1444 (9.6)	1266 (11.8)	129 (4.4)	49 (3.8)
Vital status				
Alive	6761 (45.0)	4903 (45.5)	1250 (42.4)	608 (47.1)
Dead of breast cancer	7319 (48.8)	5228 (48.5)	1478 (50.1)	613 (47.5)
Dead of other cause	932 (6.2)	643 (6.0)	220 (7.5)	69 (5.3)
Radiation therapy				
None/unknown	10246 (68.3)	7673 (71.2)	1896 (64.3)	677 (52.5)
Yes	4766 (31.7)	3101 (28.8)	1052 (35.7)	613 (47.5)
Non-primary surgical procedure to distant site				
No	14409 (96.0)	10358 (96.1)	2811 (95.4)	1240 (96.1)
Yes	475 (3.2)	66 (0.6)	40 (1.4)	22 (1.7)
Unknown	128 (0.9)	350 (3.2)	97 (3.3)	28 (2.2)
Surgical procedure				
Partial mastectomy			1111 (37.7)	249 (19.3)
Subcutaneous/simple mastectomy			707 (24.0)	416 (32.2)
Modified radical mastectomy			1054 (35.8)	574 (44.5)
(Extended) radical mastectomy			34 (1.2)	19 (1.5)
Unknown			42 (1.4)	32 (2.5)

Abbreviations: IQR, interquartile range; IDC, invasive ductal carcinoma; ILC, invasive lobular carcinoma; HR, hormone receptor; HER2, human epidermal growth factor receptor 2. *P*-value for comparison of categorical variables across groups is 0.03 for marital status, 0.001 for vital status, and <0.001 for other variables.

**Table 2:** A logistic model predicting the type of surgical procedure (*N* = 4238).

Surgical procedure	Treatment sequence	OR (95% CI)	*P-*value
Subcutaneous/simple mastectomy	Systemic therapy after surgery	1 [Reference]	<0.001
	Systemic therapy before surgery	2.32 (1.97–2.74)	

Modified radical mastectomy	Systemic therapy after surgery	1 [Reference]	<0.001
	Systemic therapy before surgery	2.23 (1.90–2.63)	

(Extended) radical mastectomy	Systemic therapy after surgery	1 [Reference]	<0.001
	Systemic therapy before surgery	2.27 (2.22–2.31)	

Only patients who underwent combined treatment with systemic therapy and surgery of the primary site were included in this model. The logistic regression model was adjusted for year of diagnosis, age, grade, *T* category, *N* category, and number of sites of metastasis. The variables above were selected using the stepwise AIC method in both directions. The reference category of surgical procedure is partial mastectomy. Abbreviations: OR, odds ratio; CI, confidence interval.

**Table 3:** Univariate and multivariate analysis of BCSD: a competing risk regression model.

Variables	Univariate analysis		Multivariate analysis	
	SHR (95% CI)	*P-*value	SHR (95% CI)	*P-*value
Treatment modality				
Systemic therapy without primary surgery	1 [Reference]	NA	1 [Reference]	NA
Systemic therapy after primary surgery	0.73 (0.69–0.77)	<0.001	0.74 (0.69–0.79)	<0.001
Systemic therapy before primary surgery	0.67 (0.62–0.73)	<0.001	0.62 (0.56–0.67)	<0.001
Treatment modality (reference category changed)				
Systemic therapy without primary surgery	—	—	—	—
Systemic therapy after primary surgery	1 [Reference]	NA	1 [Reference]	NA
Systemic therapy before primary surgery	0.92 (0.84–1.01)	0.07	0.83 (0.76–0.92)	<0.001
Year of diagnosis				
As a continuous variable	0.95 (0.94–0.96)	<0.001	0.94 (0.93–0.95)	<0.001
Age, y				
(18, 40)	1 [Reference]	NA	1 [Reference]	NA
(40, 60)	1.14 (1.04–1.24)	0.003	1.13 (1.04–1.23)	0.006
(60, 100)	1.25 (1.15–1.36)	<0.001	1.28 (1.18–1.40)	<0.001
Marital status				
Unmarried	1 [Reference]	NA	1 [Reference]	NA
Married	0.85 (0.82–0.89)	<0.001	0.91 (0.86–0.95)	<0.001
Unknown	0.95 (0.85–1.06)	0.36	0.96 (0.85–1.07)	0.46
Race				
White	1 [Reference]	NA	1 [Reference]	NA
Black	1.35 (1.27–1.43)	<0.001	1.20 (1.12–1.28)	<0.001
Other	0.94 (0.86–1.03)	0.16	0.95 (0.87–1.04)	0.27
Unknown	0.50 (0.29–0.86)	0.01	0.49 (0.28–0.85)	0.01
Sex				
Female	1 [Reference]	NA	—	—
Male	0.98 (0.79–1.2)	0.83	—	—
Histologic type				
IDC	1 [Reference]	NA	1 [Reference]	NA
ILC	0.99 (0.92–1.06)	0.70	1.25 (1.15–1.36)	<0.001
Other	1.15 (1.09–1.22)	<0.001	1.08 (1.01–1.16)	0.02
Grade				
I	1 [Reference]	NA	1 [Reference]	NA
II	1.22 (1.10–1.35)	<0.001	1.22 (1.10–1.36)	<0.001
III/IV	1.81 (1.63–2.00)	<0.001	1.63 (1.47–1.82)	<0.001
Unknown	1.66 (1.49–1.85)	<0.001	1.36 (1.22–1.53)	<0.001
AJCC *T* category				
0/1	1 [Reference]	NA	1 [Reference]	NA
2	1.08 (1.00–1.17)	0.05	1.12 (1.03–1.22)	0.008
3	1.36 (1.24–1.48)	<0.001	1.26 (1.15–1.38)	<0.001
4	1.54 (1.42–1.66)	<0.001	1.32 (1.21–1.44)	<0.001
X	1.43 (1.31–1.57)	<0.001	1.23 (1.11–1.36)	<0.001
AJCC *N* category				
0	1 [Reference]	NA	1 [Reference]	NA
1	1.03 (0.97–1.09)	0.34	0.96 (0.90–1.03)	0.27
2	1.03 (0.95–1.12)	0.42	1.04 (0.95–1.13)	0.42
3	1.18 (1.09–1.27)	<0.001	1.10 (1.01–1.19)	0.03
X	1.24 (1.12–1.37)	<0.001	0.99 (0.88–1.11)	0.83
Molecular subtype				
HR+/HER2−	1 [Reference]	NA	1 [Reference]	NA
HR+/HER2+	0.81 (0.76–0.87)	<0.001	0.70 (0.65–0.75)	<0.001
HR−/HER2+	1.02 (0.94–1.11)	0.60	0.86 (0.78–0.94)	<0.001
HR−/HER2–	2.52 (2.36–2.70)	<0.001	2.15 (1.99–2.32)	<0.001
Unknown	1.27 (1.17–1.37)	<0.001	1.06 (0.97–1.16)	0.20
Site of metastasis				
Bone only	1 [Reference]	NA	1 [Reference]	NA
Viscera only	1.23 (1.16–1.31)	<0.001	1.06 (0.99–1.14)	0.10
Bone + viscera	1.62 (1.54–1.71)	<0.001	1.09 (0.94–1.25)	0.26
Brain involvement	2.26 (2.08–2.47)	<0.001	1.38 (1.18–1.60)	<0.001
Number of sites of metastasis				
1	1 [Reference]	NA	1 [Reference]	NA
2	1.50 (1.43–1.58)	<0.001	1.33 (1.17–1.52)	<0.001
≥3	2.06 (1.91–2.22)	<0.001	1.66 (1.43–1.94)	<0.001
Radiation therapy				
None/unknown	1 [Reference]	NA	1 [Reference]	NA
Yes	1.07 (1.02–1.12)	0.005	1.06 (1.00–1.12)	0.04
Non-primary surgical procedure to distant site				
No	1 [Reference]	NA	1 [Reference]	NA
Yes	0.83 (0.73–0.96)	0.009	0.80 (0.69–0.93)	0.003
Unknown	0.79 (0.62–1.00)	0.05	0.92 (0.73–1.17)	0.52

Abbreviations: SHR, subdistribution hazard ratio; CI, confidence interval; NA, not applicable; IDC, invasive ductal carcinoma; ILC, invasive lobular carcinoma.

**Table 4:** Multivariate analysis of BCSD according to the metastatic site in patients with single-organ involvement.

	Bone-only metastasis (*N* = 6214)		Lung-only metastasis (*N* = 1725)		Liver-only metastasis (*N* = 1300)		Brain-only metastasis (*N* = 208)	
	SHR (95% CI)	*P-*value	SHR (95% CI)	*P-*value	SHR (95% CI)	*P-*value	SHR (95% CI)	*P-*value
Treatment modality in model 1								
Systemic therapy without primary surgery	1 [Reference]	NA	1 [Reference]	NA	1 [Reference]	NA	1 [Reference]	NA
Systemic therapy after primary surgery	0.66 (0.60–0.74)	<0.001	0.81 (0.68–0.97)	0.02	0.79 (0.64–0.97)	0.03	0.89 (0.57–1.39)	0.61
Systemic therapy before primary surgery	0.61 (0.53–0.69)	<0.001	0.58 (0.46–0.72)	<0.001	0.62 (0.47–0.83)	<0.001	0.64 (0.33–1.26)	0.20
Treatment modality in model 2								
Systemic therapy without primary surgery	—	—	—	—	—	—	—	—
Systemic therapy after primary surgery	1 [Reference]	NA	1 [Reference]	NA	1 [Reference]	NA	1 [Reference]	NA
Systemic therapy before primary surgery	0.92 (0.80–1.06)	0.23	0.71 (0.56–0.90)	0.005	0.79 (0.58–1.08)	0.13	0.72 (0.37–1.41)	0.34
Non-primary surgical procedure to distant site								
No	1 [Reference]	NA	1 [Reference]	NA	1 [Reference]	NA	1 [Reference]	NA
Yes	0.96 (0.75–1.22)	0.72	0.61 (0.36–1.03)	0.07	0.78 (0.46–1.33)	0.36	0.90 (0.55–1.46)	0.67
Unknown	1.34 (0.95–1.87)	0.09	0.46 (0.21–1.00)	0.05	0.55 (0.18–1.69)	0.30	0.94 (0.34–2.60)	0.90

The reference category of treatment modality on primary site varied within different models (“systemic therapy without primary surgery” for model 1 and “systemic therapy after primary surgery” for model 2). The competing risk model was adjusted for age, race, marital status, year of diagnosis, histologic type, grade, molecular subtype, AJCC *T* category, AJCC *N* category, receipt of radiotherapy, and non-primary surgical procedure to distant site. Abbreviations: SHR, subdistribution hazard ratio; CI, confidence interval; NA, not applicable.

## Data Availability

The cases were abstracted from the SEER database. Access can be obtained after submitting a request. SEER datasets and Software (RRID:SCR_003293) are available at https://seer.cancer.gov/resources/.

## Supplementary Materials

Supplementary Materials Supplementary Table 1: Multivariate analysis of all-cause death: a Cox proportional hazards model. Supplementary Table 2: Baseline characteristics of patients in the group of systemic therapy without primary surgery and the group of systemic therapy after primary surgery after propensity score matching. Supplementary Table 3: Baseline characteristics of patients in the group of systemic therapy without primary surgery and the group of systemic therapy before primary surgery after propensity score matching. Supplementary Table 4: Multivariate analysis of BCSS and OS after 1 : 1 matching the cases from the group of systemic therapy without primary surgery and the group of systemic therapy after primary surgery. Supplementary Table 5: Multivariate analysis of BCSS and OS after 1 : 1 matching the cases from the group of systemic therapy without primary surgery and the group of systemic therapy before primary surgery. Supplementary Table 6: Multivariate analysis of all-cause death according to the metastatic site in patients with single-organ involvement. Supplementary Figure 1: Kaplan–Meier curves of OS in patients with single-organ disease involving the bone (A), lung (B), liver (C), and brain (D). Supplementary Figure 2: Forest plot of subgroup analysis on BCSD, adjusted subdistribution hazard ratios. Supplementary Figure 3: Forest plot of subgroup analysis on all-cause death, adjusted hazard ratios, 15.
