# Supplementary material for: Exploring the Value of Additional Primary Tumour Excision Combined with Systemic Therapy Administered in Different Sequences for Patients with de Novo Metastatic Breast Cancer
Source: Breast J. 2022 Aug 25;2022:5049445. doi: 10.1155/2022/5049445 (PMC9436631; doi:10.1155/2022/5049445)
Supplement: Supplementary Materials — Supplementary Table 1: Multivariate analysis of all-cause death: a Cox proportional hazards model. Supplementary Table 2: Baseline characteristics of patients in the group of systemic therapy without primary surgery and the group of systemic therapy after primary surgery after propensity score matching. Supplementary Table 3: Baseline characteristics of patients in the group of systemic therapy without primary surgery and the group of systemic therapy before primary surgery after propensity score matching. Supplementary Table 4: Multivariate analysis of BCSS and OS after 1 : 1 matching the cases from the group of systemic therapy without primary surgery and the group of systemic therapy after primary surgery. Supplementary Table 5: Multivariate analysis of BCSS and OS after 1 : 1 matching the cases from the group of systemic therapy without primary surgery and the group of systemic therapy before primary surgery. Supplementary Table 6: Multivariate analysis of all-cause death according to the metastatic site in patients with single-organ involvement. Supplementary Figure 1: Kaplan–Meier curves of OS in patients with single-organ disease involving the bone (A), lung (B), liver (C), and brain (D). Supplementary Figure 2: Forest plot of subgroup analysis on BCSD, adjusted subdistribution hazard ratios. Supplementary Figure 3: Forest plot of subgroup analysis on all-cause death, adjusted hazard ratios, 15. [file 5049445.f1.zip › 5049445.f1/Supplementary content (1).docx]

**Exploring the value of additional primary tumour excision combined with systemic therapy administered in different sequences for patients with de novo metastatic breast cancer**

**Supplementary content**

[**Supplementary Table 1. Multivariate analysis of all-cause death: a Cox proportional hazards model** 2](#_Toc110274812)

[**Supplementary Table 1. (continued)** 3](#_Toc110274813)

[**Supplementary Table 2. Baseline characteristics of patients in the group of systemic therapy without primary surgery and the group of systemic therapy after primary surgery after propensity score matching** 4](#_Toc110274814)

[**Supplementary Table 2. (continued)** 5](#_Toc110274815)

[**Supplementary Table 3. Baseline characteristics of patients in the group of systemic therapy without primary surgery and the group of systemic therapy before primary surgery after propensity score matching** 6](#_Toc110274816)

[**Supplementary Table 3. (continued)** 7](#_Toc110274817)

[**Supplementary Table 4. Multivariate analysis of BCSS and OS after 1:1 matching the cases from the group of systemic therapy without primary surgery and the group of systemic therapy after primary surgery** 8](#_Toc110274818)

[**Supplementary Table 4. (continued)** 9](#_Toc110274819)

[**Supplementary Table 5. Multivariate analysis of BCSS and OS after 1:1 matching the cases from the group of systemic therapy without primary surgery and the group of systemic therapy before primary surgery** 10](#_Toc110274820)

[**Supplementary Table 5. (continued)** 11](#_Toc110274821)

[**Supplementary Table 6. Multivariate analysis of all-cause death according to the metastatic site in patients with single-organ involvement** 12](#_Toc110274822)

[**Supplementary Figure 1. Kaplan-Meier curves of OS in patients with single-organ disease involving the bone (A), lung (B), liver (C), and brain (D)** 13](#_Toc110274823)

[**Supplementary Figure 2. Forest plot of subgroup analysis on BCSD, adjusted subdistribution hazard ratios** 14](#_Toc110274824)

[**Supplementary Figure 3. Forest plot of subgroup analysis on all-cause death, adjusted hazard ratios** 15](#_Toc110274825)

**Supplementary Table 1. Multivariate analysis of all-cause death: a Cox proportional hazards model**

| Variables | HR (95% CI) | *P-value* | Overall  *P-value* |
| --- | --- | --- | --- |
| Treatment modality |  |  | < 0.001 |
| Systemic therapy without primary surgery | 1 [Reference] | NA |  |
| Systemic therapy after primary surgery | 0.74 (0.70-0.79) | < 0.001 |  |
| Systemic therapy before primary surgery | 0.57 (0.52-0.61) | < 0.001 |  |
| Treatment modality (reference category changed) | | | < 0.001 |
| Systemic therapy without primary surgery | - | - |  |
| Systemic therapy after primary surgery | 1 [Reference] | NA |  |
| Systemic therapy before primary surgery | 0.76 (0.70-0.84) | < 0.001 |  |
| Year of diagnosis |  |  | < 0.001 |
| As a continuous variable | 0.96 (0.95-0.96) | < 0.001 |  |
| Age, y |  |  |  |
| [18, 40) | 1 [Reference] | NA |  |
| [40, 60) | 1.13 (1.04-1.23) | 0.004 |  |
| [60, 100) | 1.43 (1.32-1.56) | < 0.001 |  |
| Marital status |  |  | < 0.001 |
| Unmarried | 1 [Reference] | NA |  |
| Married | 0.85 (0.81-0.89) | < 0.001 |  |
| Unknown | 0.91 (0.83-1.01) | 0.09 |  |
| Race |  |  | < 0.001 |
| White | 1 [Reference] | NA |  |
| Black | 1.26 (1.19-1.34) | < 0.001 |  |
| Other | 0.96 (0.88-1.04) | 0.34 |  |
| Unknown | 0.45 (0.26-0.78) | 0.004 |  |
| Histologic type |  |  | < 0.001 |
| IDC | 1 [Reference] | NA |  |
| ILC | 1.20 (1.11-1.30) | < 0.001 |  |
| Other | 1.11 (1.04-1.17) | 0.001 |  |
| Grade |  |  | < 0.001 |
| Ⅰ | 1 [Reference] | NA |  |
| Ⅱ | 1.17 (1.05-1.30) | 0.004 |  |
| Ⅲ/Ⅳ | 1.57 (1.41-1.75) | < 0.001 |  |
| Unknown | 1.35 (1.20-1.51) | < 0.001 |  |
| AJCC T category |  |  | < 0.001 |
| 0/1 | 1 [Reference] | NA |  |
| 2 | 1.01 (0.94-1.09) | 0.72 |  |
| 3 | 1.10 (1.01-1.20) | 0.02 |  |
| 4 | 1.23 (1.14-1.33) | < 0.001 |  |
| X | 1.15 (1.05-1.26) | 0.003 |  |

**Supplementary Table 1. (continued)**

| Variables | HR (95% CI) | *P-value* | Overall  *P-value* |
| --- | --- | --- | --- |
| AJCC N category |  |  | 0.001 |
| 0 | 1 [Reference] | NA |  |
| 1 | 0.97 (0.91-1.03) | 0.26 |  |
| 2 | 1.04 (0.96-1.13) | 0.33 |  |
| 3 | 1.09 (1.01-1.17) | 0.03 |  |
| X | 1.10 (1.00-1.20) | 0.06 |  |
| Molecular subtype |  |  | < 0.001 |
| HR+/HER2– | 1 [Reference] | NA |  |
| HR+/HER2+ | 0.66 (0.62-0.71) | < 0.001 |  |
| HR-/HER2+ | 0.84 (0.77-0.91) | < 0.001 |  |
| HR-/HER2– | 2.55 (2.39-2.72) | < 0.001 |  |
| Unknown | 1.15 (1.06-1.25) | 0.001 |  |
| Site of metastasis |  |  | < 0.001 |
| Bone only | 1 [Reference] | NA |  |
| Viscera only | 1.09 (1.03-1.17) | 0.006 |  |
| Bone + viscera | 1.09 (0.96-1.23) | 0.18 |  |
| Brain involvement | 1.46 (1.29-1.66) | < 0.001 |  |
| Number of sites of metastasis |  |  | < 0.001 |
| 1 | 1 [Reference] | NA |  |
| 2 | 1.34 (1.20-1.50) | < 0.001 |  |
| ≥ 3 | 1.74 (1.53-1.98) | < 0.001 |  |
| Radiation therapy |  |  | 0.01 |
| None/unknown | 1 [Reference] | NA |  |
| Yes | 1.06 (1.01-1.12) | 0.01 |  |
| Non-primary surgical procedure to distant site | | | < 0.001 |
| No | 1 [Reference] | NA |  |
| Yes | 0.76 (0.66-0.87) | < 0.001 |  |
| Unknown | 0.90 (0.70-1.16) | 0.42 |  |

Abbreviations: HR, hazard ratio; CI, confidence interval; NA, not applicable; IDC, invasive ductal carcinoma; ILC, invasive lobular carcinoma

**Supplementary Table 2. Baseline characteristics of patients in the group of systemic therapy without primary surgery and the group of systemic therapy after primary surgery after propensity score matching**

| Variables | Systemic therapy without primary surgery  (N =2609) | Systemic therapy after primary surgery  (N =2609) | *P-value* |
| --- | --- | --- | --- |
| Age, y |  |  | 0.83 |
| [18, 40) | 202 (7.7) | 207 (7.9) |  |
| [40, 60) | 959 (36.8) | 976 (37.4) |  |
| [60, 100) | 1448 (55.5) | 1426 (54.7) |  |
| Marital status |  |  | 0.32 |
| Unmarried | 1265 (48.5) | 1223 (46.9) |  |
| Married | 1207 (46.3) | 1260 (48.3) |  |
| Unknown | 137 (5.3) | 126 (4.8) |  |
| Race |  |  | 0.56 |
| White | 2055 (78.8) | 2021 (77.5) |  |
| Black | 363 (13.9) | 371 (14.2) |  |
| Other | 184 ( 7.1) | 210 ( 8.0) |  |
| Unknown | 7 ( 0.3) | 7 ( 0.3) |  |
| Sex |  |  |  |
| Female | 2564 (98.3) | 2563 (98.2) | 1.00 |
| Male | 45 (1.7) | 46 (1.8) |  |
| Histologic type |  |  | 0.64 |
| IDC | 1913 (73.3) | 1885 (72.2) |  |
| ILC | 291 (11.2) | 310 (11.9) |  |
| Other | 405 (15.5) | 414 (15.9) |  |
| Grade |  |  | 0.85 |
| Ⅰ | 170 ( 6.5) | 185 ( 7.1) |  |
| Ⅱ | 957 (36.7) | 963 (36.9) |  |
| Ⅲ/Ⅳ | 1240 (47.5) | 1220 (46.8) |  |
| Unknown | 242 ( 9.3) | 241 ( 9.2) |  |
| AJCC T category |  |  | 0.12 |
| 0/1 | 400 (15.3) | 437 (16.7) |  |
| 2 | 990 (37.9) | 1027 (39.4) |  |
| 3 | 465 (17.8) | 475 (18.2) |  |
| 4 | 638 (24.5) | 569 (21.8) |  |
| X | 116 ( 4.4) | 101 ( 3.9) |  |
| AJCC N category |  |  | 0.76 |
| 0 | 604 (23.2) | 614 (23.5) |  |
| 1 | 914 (35.0) | 921 (35.3) |  |
| 2 | 443 (17.0) | 408 (15.6) |  |
| 3 | 539 (20.7) | 557 (21.3) |  |
| X | 109 ( 4.2) | 109 ( 4.2) |  |

**Supplementary Table 2. (continued)**

| Variables | Systemic therapy without primary surgery  (N =2609) | Systemic therapy after primary surgery  (N =2609) | *P-value* |
| --- | --- | --- | --- |
| Subtype |  |  | 0.75 |
| HR+/HER2– | 1568 (60.1) | 1564 (59.9) |  |
| HR+/HER2+ | 191 ( 7.3) | 381 (14.6) |  |
| HR-/HER2+ | 191 ( 7.3) | 204 ( 7.8) |  |
| HR-/HER2– | 318 (12.2) | 307 (11.8) |  |
| Unknown | 136 ( 5.2) | 153 ( 5.9) |  |
| Bone involvement |  |  | 0.73 |
| No | 711 (27.3) | 723 (27.7) |  |
| Yes | 1898 (72.7) | 1886 (72.3) |  |
| Brain involvement |  |  | 0.56 |
| No | 2486 (95.3) | 2476 (94.9) |  |
| Yes | 123 ( 4.7) | 133 ( 5.1) |  |
| Liver involvement |  |  | 0.75 |
| No | 1984 (76.0) | 1973 (75.6) |  |
| Yes | 625 (24.0) | 636 (24.4) |  |
| Lung involvement |  |  | 0.28 |
| No | 1802 (69.1) | 1839 (70.5) |  |
| Yes | 807 (30.9) | 770 (29.5) |  |
| Site of metastasis |  |  | 0.90 |
| Bone only | 1289 (49.4) | 1294 (49.6) |  |
| Viscera only | 667 (25.6) | 666 (25.5) |  |
| Bone + viscera | 530 (20.3) | 516 (19.8) |  |
| Brain involvement | 123 ( 4.7) | 133 ( 5.1) |  |
| Number of sites of metastasis |  |  | 0.79 |
| 1 | 1922 (73.7) | 1932 (74.1) |  |
| 2 | 547 (21.0) | 548 (21.0) |  |
| ≥ 3 | 140 ( 5.4) | 129 ( 4.9) |  |
| Radiation therapy |  |  | 0.88 |
| None/unknown | 1730 (66.3) | 1736 (66.5) |  |
| Yes | 879 (33.7) | 873 (33.5) |  |
| Non-primary surgical procedure to distant site | | | 0.39 |
| No | 2490 (95.4) | 2488 (95.4) |  |
| Yes | 81 ( 3.1) | 92 ( 3.5) |  |
| Unknown | 38 ( 1.5) | 29 ( 1.1) |  |

Abbreviations: IDC, invasive ductal carcinoma; ILC, invasive lobular carcinoma

**Supplementary Table 3. Baseline characteristics of patients in the group of systemic therapy without primary surgery and the group of systemic therapy before primary surgery after propensity score matching**

| Variables | Systemic therapy without primary surgery  (N = 1283) | Systemic therapy  before primary surgery  (N = 1283) | *P-value* |
| --- | --- | --- | --- |
| Age, y |  |  | 0.85 |
| [18, 40) | 133 (10.4) | 141 (11.0) |  |
| [40, 60) | 661 (51.5) | 650 (50.7) |  |
| [60, 100) | 489 (38.1) | 492 (38.3) |  |
| Marital status |  |  | 0.82 |
| Unmarried | 609 (47.5) | 594 (46.3) |  |
| Married | 601 (46.8) | 617 (48.1) |  |
| Unknown | 73 ( 5.7) | 72 ( 5.6) |  |
| Race |  |  | 0.97 |
| White | 931 (72.6) | 936 (73.0) |  |
| Black | 229 (17.8) | 224 (17.5) |  |
| Other | 121 ( 9.4) | 120 ( 9.4) |  |
| Unknown | 2 ( 0.2) | 3 ( 0.2) |  |
| Sex |  |  | 0.41 |
| Female | 1273 (99.2) | 1269 (98.9) |  |
| Male | 10 ( 0.8) | 14 ( 1.1) |  |
| Histologic type |  |  | 0.68 |
| IDC | 1032 (80.4) | 1019 (79.4) |  |
| ILC | 90 ( 7.0) | 88 ( 6.9) |  |
| Other | 161 (12.5) | 176 (13.7) |  |
| Grade |  |  | 0.81 |
| Ⅰ | 83 ( 6.5) | 86 ( 6.7) |  |
| Ⅱ | 413 (32.2) | 414 (32.3) |  |
| Ⅲ/Ⅳ | 673 (52.5) | 656 (51.1) |  |
| Unknown | 114 ( 8.9) | 127 ( 9.9) |  |
| AJCC T category |  |  | 0.92 |
| 0/1 | 124 ( 9.7) | 117 ( 9.1) |  |
| 2 | 338 (26.3) | 355 (27.7) |  |
| 3 | 338 (26.3) | 237 (18.5) |  |
| 4 | 518 (40.4) | 507 (39.5) |  |
| X | 62 ( 4.8) | 67 ( 5.2) |  |
| AJCC N category |  |  | 0.89 |
| 0 | 184 (14.3) | 202 (15.7) |  |
| 1 | 605 (47.2) | 597 (46.5) |  |
| 2 | 209 (16.3) | 208 (16.2) |  |
| 3 | 238 (18.6) | 233 (18.2) |  |
| X | 47 ( 3.7) | 43 ( 3.4) |  |

**Supplementary Table 3. (continued)**

| Variables | Systemic therapy without primary surgery  (N = 1283) | Systemic therapy  before primary surgery  (N = 1283) | *P-value* |
| --- | --- | --- | --- |
| Subtype |  |  | 0.70 |
| HR+/HER2– | 612 (47.7) | 605 (47.2) |  |
| HR+/HER2+ | 235 (18.3) | 213 (16.6) |  |
| HR-/HER2+ | 127 ( 9.9) | 141 (11.0) |  |
| HR-/HER2– | 240 (18.7) | 251 (19.6) |  |
| Unknown | 69 ( 5.4) | 73 ( 5.7) |  |
| Bone involvement |  |  | 0.50 |
| No | 425 (33.1) | 442 (34.5) |  |
| Yes | 858 (66.9) | 841 (65.5) |  |
| Brain involvement |  |  | 0.92 |
| No | 1230 (95.9) | 1229 (95.8) |  |
| Yes | 53 ( 4.1) | 54 ( 4.2) |  |
| Liver involvement |  |  | 0.96 |
| No | 1003 (78.2) | 1002 (78.1) |  |
| Yes | 280 (21.8) | 281 (21.9) |  |
| Lung involvement |  |  | 0.80 |
| No | 872 (68.2) | 865 (67.4) |  |
| Yes | 411 (32.0) | 418 (32.6) |  |
| Site of metastasis |  |  | 0.91 |
| Bone only | 627 (48.9) | 617 (48.1) |  |
| Viscera only | 392 (30.6) | 408 (31.8) |  |
| Bone + viscera | 211 (16.4) | 204 (15.9) |  |
| Brain involvement | 53 ( 4.1) | 54 ( 4.2) |  |
| Number of sites of metastasis |  |  | 0.87 |
| 1 | 1014 (79.0) | 1023 (79.7) |  |
| 2 | 221 (17.2) | 211 (16.4) |  |
| ≥ 3 | 48 ( 3.7) | 48 ( 3.7) |  |
| Radiation therapy |  |  | 0.97 |
| None/unknown | 678 (52.8) | 677 (52.8) |  |
| Yes | 605 (47.2) | 606 (47.2) |  |
| Non-primary surgical procedure to distant site | | | 0.50 |
| No | 1225 (95.5) | 1236 (96.3) |  |
| Yes | 37 ( 2.9) | 28 ( 2.2) |  |
| Unknown | 21 ( 1.6) | 19 ( 1.5) |  |

Abbreviations: IDC, invasive ductal carcinoma; ILC, invasive lobular carcinoma

**Supplementary Table 4. Multivariate analysis of BCSS and OS after 1:1 matching the cases from the group of systemic therapy without primary surgery and the group of systemic therapy after primary surgery**

| Variables | Multivariate Analysis of BCSS | | Multivariate Analysis of OS | |
| --- | --- | --- | --- | --- |
|  | SHR (95% CI) | *P-value* | HR (95% CI) | *P-value* |
| Treatment modality |  |  |  |  |
| Systemic therapy without primary surgery | 1 [Reference] | NA | 1 [Reference] | NA |
| Systemic therapy after primary surgery | 0.75 (0.69-0.82) | < 0.001 | 0.74 (0.68-0.80) | < 0.001 |
| Year of diagnosis |  |  |  |  |
| As a continuous variable | 0.94 (0.93-0.96) | < 0.001 | 0.96 (0.94-0.97) | < 0.001 |
| Age, y |  |  |  |  |
| [18, 40) | 1 [Reference] | NA | 1 [Reference] | NA |
| [40, 60) | 1.30 (1.12-1.52) | 0.03 | 1.16 (1.00-1.36) | 0.05 |
| [60, 100) | 1.19 (1.02-1.38) | 0.001 | 1.48 (1.27-1.72) | < 0.001 |
| Marital status |  |  |  |  |
| Unmarried | 1 [Reference] | NA | 1 [Reference] | NA |
| Married | 0.94 (0.86-1.02) | 0.13 | 0.86 (0.79-0.92) | < 0.001 |
| Unknown | 1.03 (0.85-1.25) | 0.74 | 1.01 (0.85-1.20) | 0.88 |
| Race |  |  |  |  |
| White | 1 [Reference] | NA | 1 [Reference] | NA |
| Black | 1.13 (1.00-1.27) | 0.05 | 1.20 (1.08-1.33) | 0.001 |
| Other | 0.96 (0.81-1.13) | 0.60 | 0.98 (0.85-1.14) | 0.82 |
| Unknown | 0.94 (0.43-2.08) | 0.88 | 0.72 (0.32-1.61) | 0.42 |
| Histologic type |  |  |  |  |
| IDC | 1 [Reference] | NA | 1 [Reference] | NA |
| ILC | 1.27 (1.12-1.45) | < 0.001 | 1.20 (1.06-1.36) | 0.005 |
| Other | 1.10 (0.98-1.23) | 0.10 | 1.10 (1.00-1.22) | 0.06 |
| Grade |  |  |  |  |
| Ⅰ | 1 [Reference] | NA | 1 [Reference] | NA |
| Ⅱ | 1.25 (1.05-1.49) | 0.01 | 1.21 (1.02-1.44) | 0.03 |
| Ⅲ/Ⅳ | 1.78 (1.49-2.13) | < 0.001 | 1.72 (1.45-2.05) | < 0.001 |
| Unknown | 1.49 (1.19-1.87) | 0.001 | 1.45 (1.17-1.78) | 0.001 |
| AJCC T category |  |  |  |  |
| 0/1 | 1 [Reference] | NA | 1 [Reference] | NA |
| 2 | 1.02 (0.90-1.17) | 0.73 | 1.00 (0.89-1.13) | 0.96 |
| 3 | 1.24 (1.07-1.44) | 0.004 | 1.20 (1.05-1.37) | 0.009 |
| 4 | 1.29 (1.12-1.49) | < 0.001 | 1.36 (1.19-1.55) | < 0.001 |
| X | 1.05 (0.82-1.35) | 0.70 | 1.09 (0.87-1.35) | 0.47 |
| AJCC N category |  |  |  |  |
| 0 | 1 [Reference] | NA | 1 [Reference] | NA |
| 1 | 0.95 (0.85-1.07) | 0.40 | 0.99 (0.89-1.10) | 0.86 |
| 2 | 0.99 (0.86-1.14) | 0.89 | 1.03 (0.91-1.17) | 0.65 |
| 3 | 1.06 (0.93-1.20) | 0.37 | 1.10 (0.98-1.24) | 0.10 |
| X | 0.96 (0.74-1.24) | 0.75 | 1.27 (1.04-1.54) | 0.02 |

**Supplementary Table 4. (continued)**

| Variables | Multivariate Analysis of BCSS | | Multivariate Analysis of OS | |
| --- | --- | --- | --- | --- |
|  | SHR (95% CI) | *P-value* | SHR (95% CI) | *P-value* |
| Subtype |  |  |  |  |
| HR+/HER2– | 1 [Reference] | NA | 1 [Reference] | NA |
| HR+/HER2+ | 0.66 (0.58-0.75) | < 0.001 | 0.64 (0.57-0.72) | < 0.001 |
| HR-/HER2+ | 0.81 (0.68-0.96) | 0.01 | 0.78 (0.67-0.91) | 0.001 |
| HR-/HER2– | 2.20 (1.92-2.53) | < 0.001 | 2.58 (2.31-2.89) | < 0.001 |
| Unknown | 1.13 (0.95-1.35) | 0.17 | 1.23 (1.05-1.44) | 0.01 |
| Site of metastasis |  |  |  |  |
| Bone only | 1 [Reference] | NA | 1 [Reference] | NA |
| Viscera only | 1.17 (1.04-1.31) | 0.008 | 1.16 (1.05-1.28) | 0.004 |
| Bone + viscera | 1.35 (1.05-1.73) | 0.02 | 1.20 (0.97-1.48) | 0.10 |
| Brain involvement | 1.52 (1.16-1.99) | 0.003 | 1.58 (1.26-1.98) | < 0.001 |
| Number of sites of metastasis |  |  |  |  |
| 1 | 1 [Reference] | NA | 1 [Reference] | NA |
| 2 | 1.16 (0.92-1.45) | 0.21 | 1.28 (1.05-1.55) | 0.01 |
| ≥ 3 | 1.35 (1.02-1.79) | 0.04 | 1.45 (1.14-1.85) | 0.002 |
| Radiation therapy |  |  |  |  |
| None/unknown | 1 [Reference] | NA | 1 [Reference] | NA |
| Yes | 1.02 (0.94-1.12) | 0.60 | 1.03 (0.95-1.12) | 0.47 |
| Non-primary surgical procedure to distant site | | | |  |
| No | 1 [Reference] | NA | 1 [Reference] | NA |
| Yes | 0.85 (0.67-1.08) | 0.18 | 0.79 (0.63-0.99) | 0.04 |
| Unknown | 0.85 (0.59-1.21) | 0.36 | 0.79 (0.55-1.12) | 0.19 |

Abbreviations: BCSS, breast cancer-specific survival; OS, overall survival; SHR, subdistribution hazard ratio; CI, confidence interval; IDC, invasive ductal carcinoma; ILC, invasive lobular carcinoma

**Supplementary Table 5. Multivariate analysis of BCSS and OS after 1:1 matching the cases from the group of systemic therapy without primary surgery and the group of systemic therapy before primary surgery**

| Variables | Multivariate Analysis of BCSS | | Multivariate Analysis of OS | |
| --- | --- | --- | --- | --- |
|  | SHR (95% CI) | *P-value* | HR (95% CI) | *P-value* |
| Treatment modality |  |  |  |  |
| Systemic therapy without primary surgery | 1 [Reference] | NA | 1 [Reference] | NA |
| Systemic therapy before primary surgery | 0.55 (0.49-0.62) | < 0.001 | 0.60 (0.53-0.68) | < 0.001 |
| Year of diagnosis |  |  |  |  |
| As a continuous variable | 0.97 (0.94-0.99) | 0.005 | 0.95 (0.93-0.98) | < 0.001 |
| Age, y |  |  |  |  |
| [18, 40) | 1 [Reference] | NA | 1 [Reference] | NA |
| [40, 60) | 0.92 (0.77-1.11) | 0.39 | 0.96 (0.79-1.17) | 0.69 |
| [60, 100) | 1.19 (0.99-1.44) | 0.07 | 1.21 (0.99-1.48) | 0.06 |
| Marital status |  |  |  |  |
| Unmarried | 1 [Reference] | NA | 1 [Reference] | NA |
| Married | 0.84 (0.75-0.94) | 0.003 | 0.91 (0.80-1.03) | 0.12 |
| Unknown | 0.86 (0.67-1.10) | 0.24 | 0.89 (0.68-1.16) | 0.38 |
| Race |  |  |  |  |
| White | 1 [Reference] | NA | 1 [Reference] | NA |
| Black | 1.35 (1.18-1.55) | < 0.001 | 1.30 (1.11-1.52) | 0.009 |
| Other/ Unknown | 0.92 (0.75-1.13) | 0.44 | 1.00 (0.81-1.24) | 0.98 |
| Histologic type |  |  |  |  |
| IDC | 1 [Reference] | NA | 1 [Reference] | NA |
| ILC | 1.26 (1.02-1.57) | 0.04 | 1.24 (0.98-1.56) | 0.07 |
| Other | 1.20 (1.02-1.41) | 0.03 | 1.20 (1.01-1.42) | 0.04 |
| Grade |  |  |  |  |
| Ⅰ | 1 [Reference] | NA | 1 [Reference] | NA |
| Ⅱ | 1.07 (0.83-1.38) | 0.60 | 1.09 (0.85-1.39) | 0.50 |
| Ⅲ/Ⅳ | 1.51 (1.17-1.96) | 0.002 | 1.50 (1.17-1.92) | 0.002 |
| Unknown | 1.28 (0.94-1.72) | 0.11 | 1.08 (0.79-1.48) | 0.63 |
| AJCC T category |  |  |  |  |
| 0/1 | 1 [Reference] | NA | 1 [Reference] | NA |
| 2 | 1.19 (0.95-1.50) | 0.13 | 1.40 (1.09-1.80) | 0.009 |
| 3 | 1.22 (0.96-1.54) | 0.11 | 1.43 (1.09-1.86) | 0.009 |
| 4 | 1.55 (1.24-1.94) | < 0.001 | 1.70 (1.32-2.18) | < 0.001 |
| X | 1.22 (0.88-1.69) | 0.23 | 1.41 (1.00-1.98) | 0.05 |
| AJCC N category |  |  |  |  |
| 0 | 1 [Reference] | NA | 1 [Reference] | NA |
| 1 | 1.02 (0.87-1.21) | 0.78 | 1.03 (0.86-1.23) | 0.78 |
| 2 | 1.12 (0.92-1.36) | 0.26 | 1.16 (0.94-1.44) | 0.18 |
| 3 | 1.16 (0.96-1.41) | 0.12 | 1.18 (0.95-1.46) | 0.14 |
| X | 1.18 (0.86-1.64) | 0.31 | 1.10 (0.74-1.62) | 0.64 |

**Supplementary Table 5. (continued)**

| Variables | Multivariate Analysis of BCSS | | Multivariate Analysis of OS | |
| --- | --- | --- | --- | --- |
|  | SHR (95% CI) | *P-value* | SHR (95% CI) | *P-value* |
| Subtype |  |  |  |  |
| HR+/HER2– | 1 [Reference] | NA | 1 [Reference] | NA |
| HR+/HER2+ | 0.64 (0.54-0.76) | < 0.001 | 0.67 (0.56-0.79) | < 0.001 |
| HR-/HER2+ | 0.73 (0.59-0.89) | 0.002 | 0.65 (0.52-0.83) | < 0.001 |
| HR-/HER2– | 2.30 (1.99-2.67) | < 0.001 | 2.00 (1.70-2.36) | < 0.001 |
| Unknown | 0.98 (0.78-1.23) | 0.84 | 0.95 (0.74-1.21) | 0.68 |
| Site of metastasis |  |  |  |  |
| Bone only | 1 [Reference] | NA | 1 [Reference] | NA |
| Viscera only | 1.00 (0.87-1.14) | 0.98 | 1.00 (0.87-1.16) | 0.97 |
| Bone + viscera | 1.19 (0.84-1.67) | 0.33 | 1.09 (0.73-1.64) | 0.68 |
| Brain involvement | 1.44 (1.04-1.98) | 0.03 | 1.44 (0.95-2.19) | 0.09 |
| Number of sites of metastasis |  |  |  |  |
| 1 | 1 [Reference] | NA | 1 [Reference] | NA |
| 2 | 1.23 (0.90-1.68) | 0.19 | 1.26 (0.87-1.84) | 0.23 |
| ≥ 3 | 2.40 (1.65-3.51) | < 0.001 | 2.29 (1.45-3.63) | < 0.001 |
| Radiation therapy |  |  |  |  |
| None/unknown | 1 [Reference] | NA | 1 [Reference] | NA |
| Yes | 1.07 (0.96-1.19) | 0.24 | 1.08 (0.95-1.22) | 0.24 |
| Non-primary surgical procedure to distant site | | | |  |
| No | 1 [Reference] | NA | 1 [Reference] | NA |
| Yes | 0.57 (0.39-0.84) | 0.005 | 0.59 (0.37-0.92) | 0.02 |
| Unknown | 0.69 (0.41-1.18) | 0.18 | 0.77 (0.44-1.33) | 0.34 |

Abbreviations: BCSS, breast cancer-specific survival; OS, overall survival; SHR, subdistribution hazard ratio; CI, confidence interval; IDC, invasive ductal carcinoma; ILC, invasive lobular carcinoma

**Supplementary Table 6. Multivariate analysis of all-cause death according to the metastatic site in patients with single-organ involvement**

|  | Bone-only metastasis | | Lung-only metastasis | | Liver-only metastasis | | Brain-only metastasis | |
| --- | --- | --- | --- | --- | --- | --- | --- | --- |
|  | HR (95% CI) | P-value | HR (95% CI) | P-value | HR (95% CI) | P-value | HR (95% CI) | P-value |
| Treatment modality in Model 1 |  |  |  |  |  |  |  |  |
| Systemic therapy without primary surgery | 1 [Reference] | NA | 1 [Reference] | NA | 1 [Reference] | NA | 1 [Reference] | NA |
| Systemic therapy after primary surgery | 0.68 (0.62-0.75) | < 0.001 | 0.77 (0.65-0.92) | 0.003 | 0.81 (0.67-0.99) | 0.04 | 0.77 (0.49-1.24) | 0.28 |
| Systemic therapy before primary surgery | 0.56 (0.49-0.64) | < 0.001 | 0.54 (0.44-0.66) | < 0.001 | 0.59 (0.45-0.79) | < 0.001 | 0.70 (0.27-1.31) | 0.26 |
| Treatment modality in Model 2 |  |  |  |  |  |  |  |  |
| Systemic therapy without primary surgery | - | - | - | - | - | - | - | - |
| Systemic therapy after primary surgery | 1 [Reference] | NA | 1 [Reference] | NA | 1 [Reference] | NA | 1 [Reference] | NA |
| Systemic therapy before primary surgery | 0.83 (0.72-0.95) | 0.008 | 0.70 (0.55-0.88) | 0.002 | 0.73 (0.54-1.00) | 0.05 | 0.90 (0.46-1.75) | 0.75 |
| Non-primary surgical procedure to distant site |  |  |  |  |  |  |  |  |
| No | 1 [Reference] | NA | 1 [Reference] | NA | 1 [Reference] | NA | 1 [Reference] | NA |
| Yes | 0.97 (0.78-1.23) | 0.83 | 0.47 (0.29-0.75) | 0.002 | 0.73 (0.43-1.26) | 0.26 | 0.84 (0.51-1.40) | 0.51 |
| Unknown | 1.29 (0.90-1.84) | 0.16 | 0.46 (0.21-1.04) | 0.06 | 0.48 (0.12-1.95) | 0.30 | 0.41 (0.05-3.17) | 0.40 |

The reference category of treatment modality on primary site varied within different models (“systemic therapy without primary surgery” for Model 1 and “systemic therapy after primary surgery” for Model 2). The Cox regression model was adjusted for age, race, marital status, year of diagnosis, histologic type, grade, molecular subtype, AJCC T category, AJCC N category, receipt of radiotherapy, and non-primary surgical procedure to distant site.

Abbreviations: HR, hazard ratio; CI, confidence interval; NA, not applicable

**
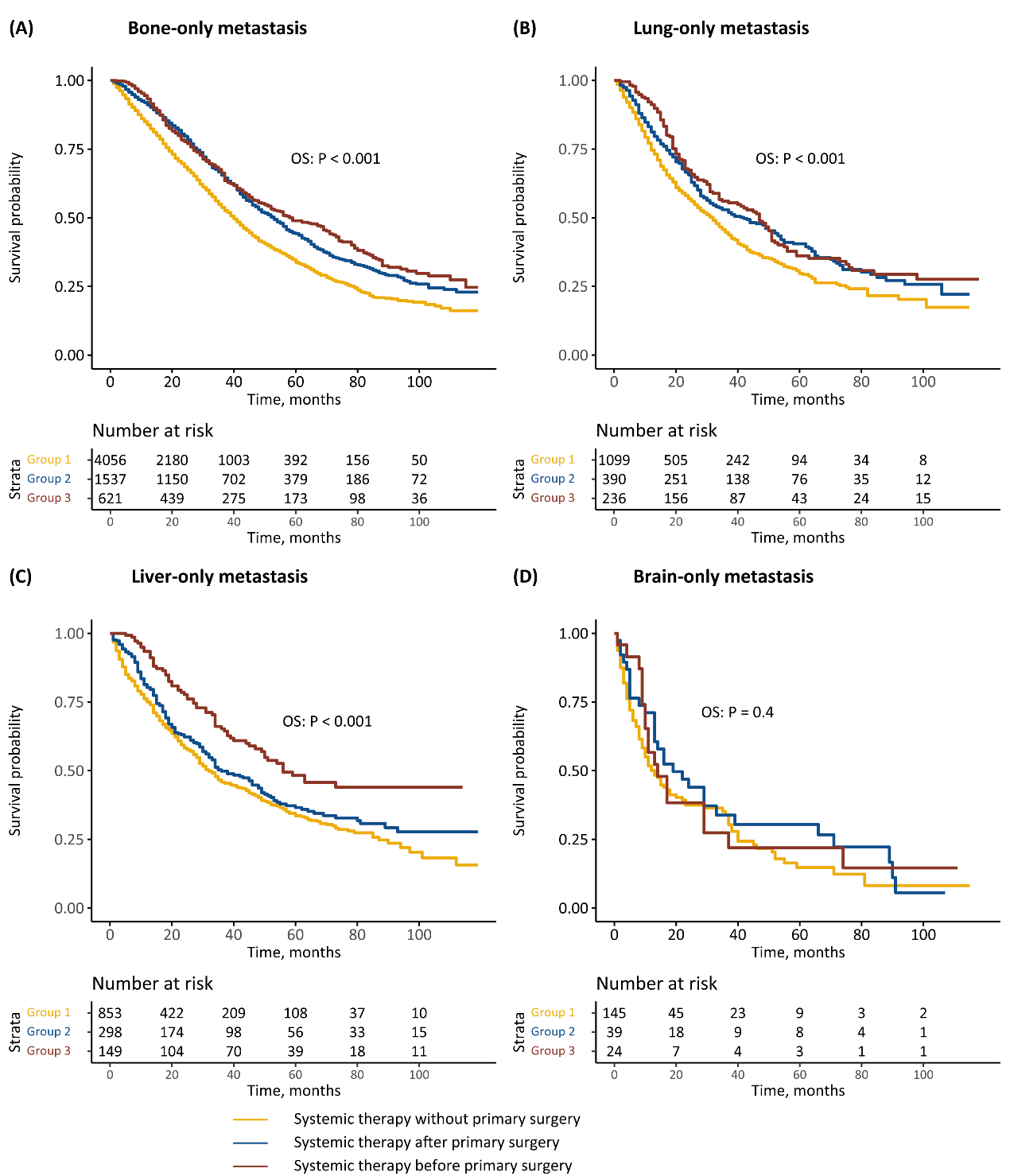
**

**Supplementary Figure 1. Kaplan-Meier curves of OS in patients with single-organ disease involving the bone (A), lung (B), liver (C), and brain (D)**


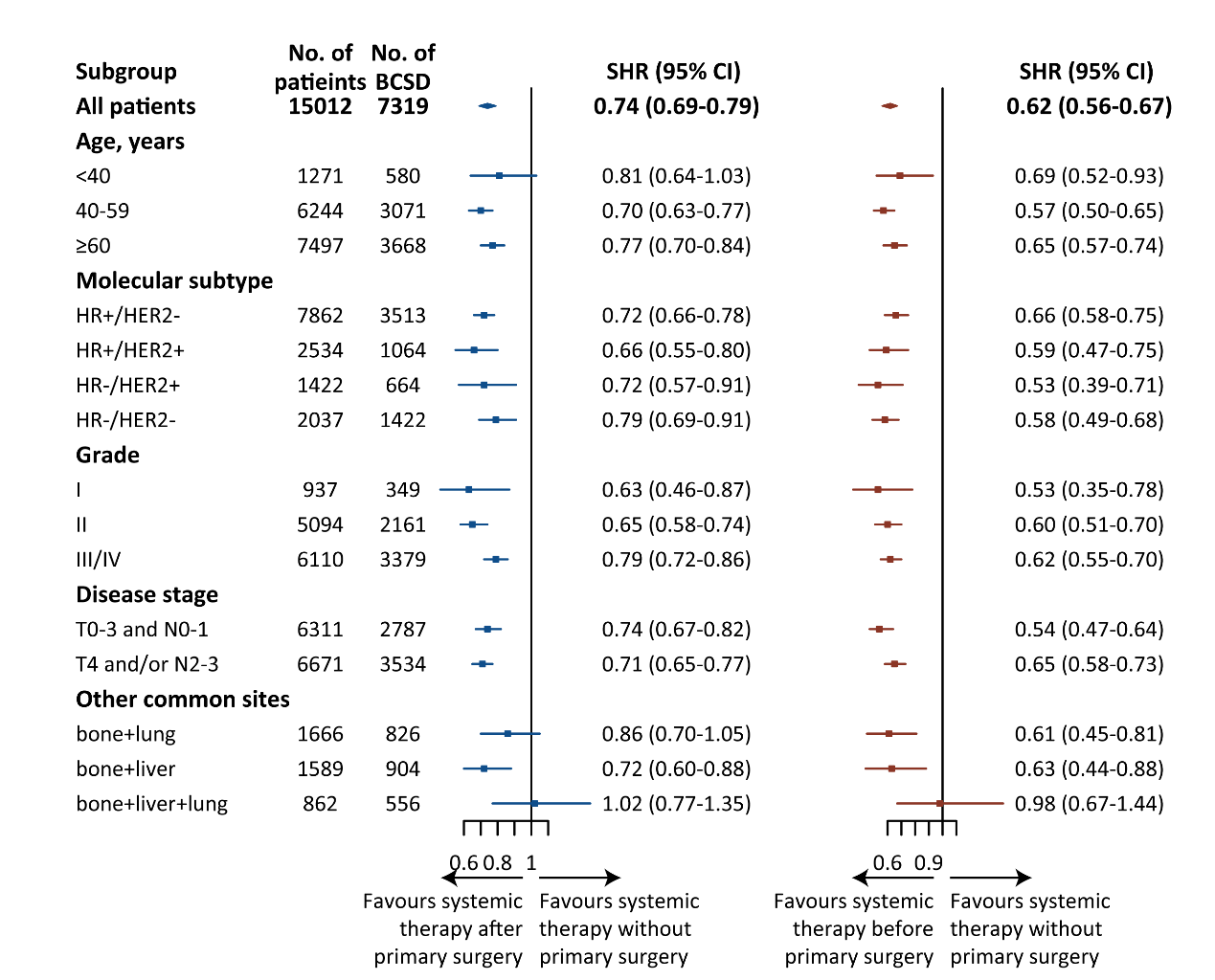


**Supplementary Figure 2. Forest plot of subgroup analysis on BCSD, adjusted subdistribution hazard ratios**

The Fine-Grey regression model was adjusted for age, race, marital status, year of diagnosis, histologic type, grade, molecular subtype, disease stage, category of metastatic sites, number of sites of metastasis, receipt of radiotherapy, and non-primary surgical procedure to distant site (The variable representing the subgroup was exculded from analysis of each subgroup).

Abbreviations: BCSD, breast cancer-specific death; SHR, subdistribution hazard ratio; CI, confidence interval

**
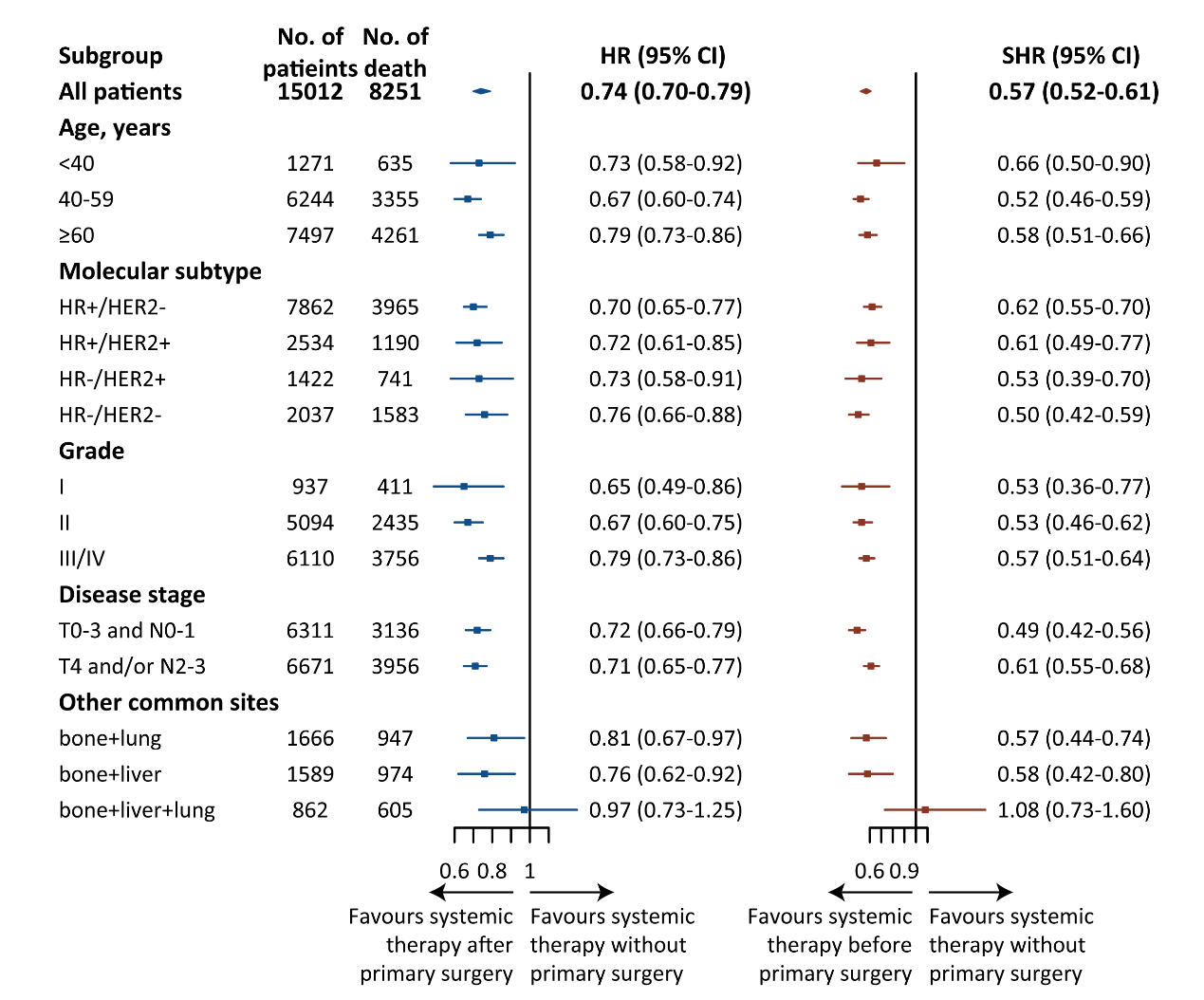
**

**Supplementary Figure 3. Forest plot of subgroup analysis on all-cause death, adjusted hazard ratios**

The Cox regression model was adjusted for age, race, marital status, year of diagnosis, histologic type, grade, molecular subtype, disease stage, category of metastatic sites, number of sites of metastasis, receipt of radiotherapy, and non-primary surgical procedure to distant site (The variable representing the subgroup was exculded from analysis of each subgroup).

Abbreviations: HR, hazard ratio; CI, confidence interval
