# Supplementary material for: Exploring the Value of Additional Primary Tumour Excision Combined with Systemic Therapy Administered in Different Sequences for Patients with de Novo Metastatic Breast Cancer
Source: Breast J. 2022 Aug 25;2022:5049445. doi: 10.1155/2022/5049445 (PMC9436631; doi:10.1155/2022/5049445)
Supplement: Supplementary Materials — Supplementary Table 1: Multivariate analysis of all-cause death: a Cox proportional hazards model. Supplementary Table 2: Baseline characteristics of patients in the group of systemic therapy without primary surgery and the group of systemic therapy after primary surgery after propensity score matching. Supplementary Table 3: Baseline characteristics of patients in the group of systemic therapy without primary surgery and the group of systemic therapy before primary surgery after propensity score matching. Supplementary Table 4: Multivariate analysis of BCSS and OS after 1 : 1 matching the cases from the group of systemic therapy without primary surgery and the group of systemic therapy after primary surgery. Supplementary Table 5: Multivariate analysis of BCSS and OS after 1 : 1 matching the cases from the group of systemic therapy without primary surgery and the group of systemic therapy before primary surgery. Supplementary Table 6: Multivariate analysis of all-cause death according to the metastatic site in patients with single-organ involvement. Supplementary Figure 1: Kaplan–Meier curves of OS in patients with single-organ disease involving the bone (A), lung (B), liver (C), and brain (D). Supplementary Figure 2: Forest plot of subgroup analysis on BCSD, adjusted subdistribution hazard ratios. Supplementary Figure 3: Forest plot of subgroup analysis on all-cause death, adjusted hazard ratios, 15. [file 5049445.f1.zip › 5049445.f1/Supplementary Figure 1. Kaplan-Meier curves of OS in patients with single-organ disease involving the bone (A), lung (B), liver (C), and brain (D) (2).pdf]

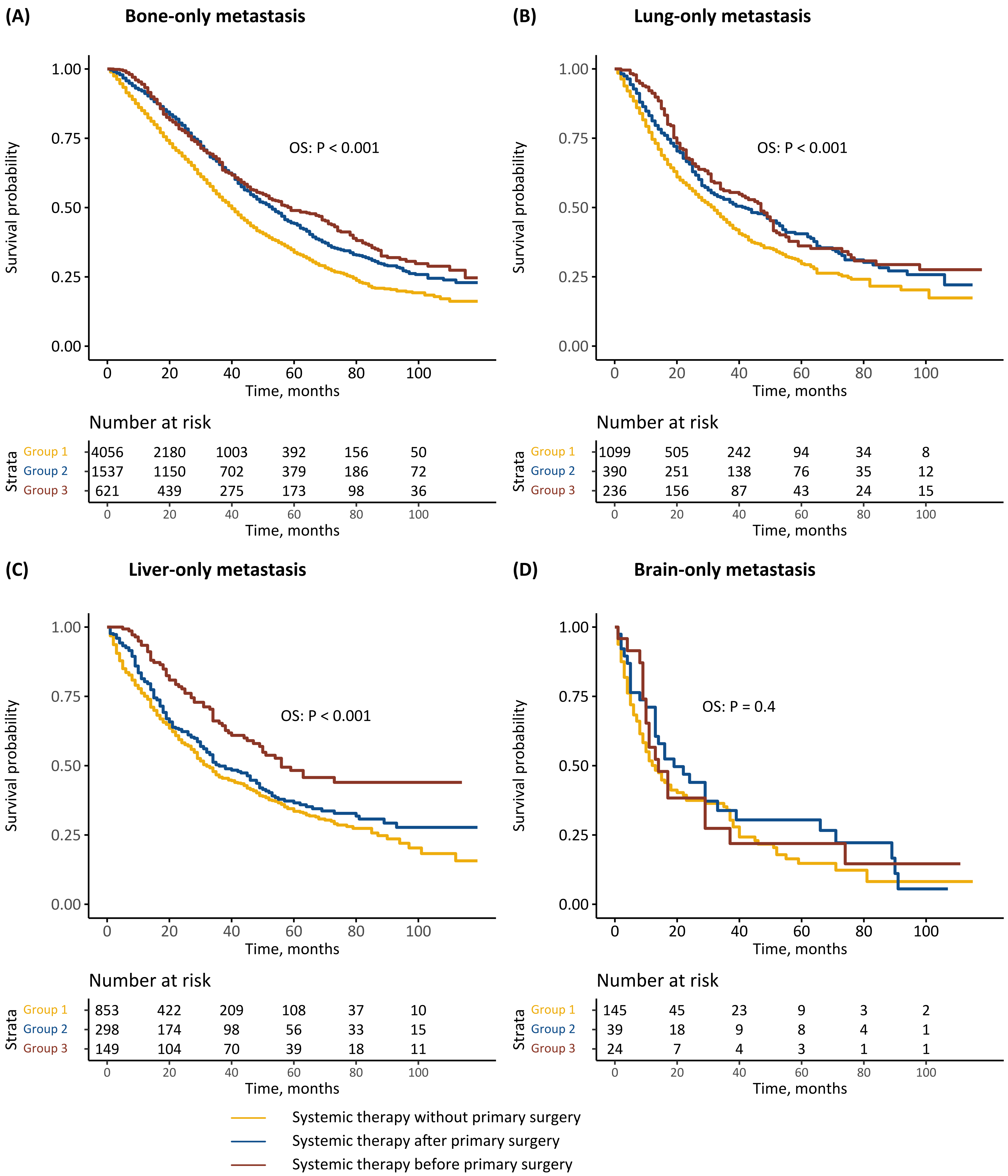
(A)
Bone-only metastasis
Survival probability
1.00
0.75
0.50
0.25
0.00
OS: P < 0.001
0 20 40 60 80 100
Time, months
Number at risk
Strata
Group 1 4056 2180 1003 392 156 50
Group 2 1537 1150 702 379 186 72
Group 3 621 439 275 173 98 36
0 20 40 60 80 100
Time, months
(B)
Lung-only metastasis
Survival probability
1.00
0.75
0.50
0.25
0.00
OS: P < 0.001
0 20 40 60 80 100
Time, months
Number at risk
Strata
Group 1 1099 505 242 94 34 8
Group 2 390 251 138 76 35 12
Group 3 236 156 87 43 24 15
0 20 40 60 80 100
Time, months
(C)
Liver-only metastasis
Survival probability
1.00
0.75
0.50
0.25
0.00
OS: P < 0.001
0 20 40 60 80 100
Time, months
Number at risk
Strata
Group 1 853 422 209 108 37 10
Group 2 298 174 98 56 33 15
Group 3 149 104 70 39 18 11
0 20 40 60 80 100
Time, months
(D)
Brain-only metastasis
Survival probability
1.00
0.75
0.50
0.25
0.00
OS: P = 0.4
0 20 40 60 80 100
Time, months
Number at risk
Strata
Group 1 145 45 23 9 3 2
Group 2 39 18 9 8 4 1
Group 3 24 7 4 3 1 1
0 20 40 60 80 100
Time, months
Systemic therapy without primary surgery
Systemic therapy after primary surgery
Systemic therapy before primary surgery
