# Supplementary material for: Exploring the Value of Additional Primary Tumour Excision Combined with Systemic Therapy Administered in Different Sequences for Patients with de Novo Metastatic Breast Cancer
Source: Breast J. 2022 Aug 25;2022:5049445. doi: 10.1155/2022/5049445 (PMC9436631; doi:10.1155/2022/5049445)
Supplement: Supplementary Materials — Supplementary Table 1: Multivariate analysis of all-cause death: a Cox proportional hazards model. Supplementary Table 2: Baseline characteristics of patients in the group of systemic therapy without primary surgery and the group of systemic therapy after primary surgery after propensity score matching. Supplementary Table 3: Baseline characteristics of patients in the group of systemic therapy without primary surgery and the group of systemic therapy before primary surgery after propensity score matching. Supplementary Table 4: Multivariate analysis of BCSS and OS after 1 : 1 matching the cases from the group of systemic therapy without primary surgery and the group of systemic therapy after primary surgery. Supplementary Table 5: Multivariate analysis of BCSS and OS after 1 : 1 matching the cases from the group of systemic therapy without primary surgery and the group of systemic therapy before primary surgery. Supplementary Table 6: Multivariate analysis of all-cause death according to the metastatic site in patients with single-organ involvement. Supplementary Figure 1: Kaplan–Meier curves of OS in patients with single-organ disease involving the bone (A), lung (B), liver (C), and brain (D). Supplementary Figure 2: Forest plot of subgroup analysis on BCSD, adjusted subdistribution hazard ratios. Supplementary Figure 3: Forest plot of subgroup analysis on all-cause death, adjusted hazard ratios, 15. [file 5049445.f1.zip › 5049445.f1/Tables (1).docx]

Table 1. Demographic, clinicopathologic, and treatment characteristics of the included patients

| Variables | All patients  (N = 15012) | Systemic therapy without primary surgery  (N = 10774) | Systemic therapy after primary surgery  (N = 2948) | Systemic therapy before primary surgery  (N = 1290 ) |
| --- | --- | --- | --- | --- |
| Age, y |  |  |  |  |
| [18, 40) | 1271 (8.5) | 907 (8.4) | 221 (7.5) | 143 (11.1) |
| [40, 60) | 6244 (41.6) | 4511 (41.9) | 1079 (36.6) | 654 (50.7) |
| [60, 100) | 7497 (49.9) | 5356 (49.7) | 1648 (55.9) | 493 (38.2) |
| Median (IQR) | 59 (50-68) | 59 (50-68) | 62 (51-71) | 56 (47-64) |
| Marital status |  |  |  |  |
| Unmarried | 7199 (48.0) | 5244 (48.7) | 1359 (46.1) | 596 (46.2) |
| Married | 7125 (47.5) | 5058 (46.9) | 1445 (49.0) | 622 (48.2) |
| Unknown | 688 (4.6) | 472 (4.4) | 144 (4.9) | 72 (5.6) |
| Race |  |  |  |  |
| White | 11197 (74.6) | 7938 (73.7) | 2316 (78.6) | 943 (73.1) |
| Black | 2415 (16.1) | 1794 (16.7) | 397 (13.5) | 224 (17.4) |
| Other | 1333 (8.9) | 985 (9.1) | 228 (7.7) | 120 (9.3) |
| Unknown | 67 (0.4) | 57 (0.5) | 7 (0.2) | 3 (0.2) |
| Sex |  |  |  |  |
| Female | 14831 (98.8) | 10666 (9.0) | 2889 (98.0) | 1276 (98.9) |
| Male | 181 (1.2) | 108 (1.0) | 59 (2.0) | 14 (1.1) |
| Histologic type |  |  |  |  |
| IDC | 10644 (70.9) | 7494 (69.6) | 2125 (72.1) | 1025 (79.5) |
| ILC | 1499 (10.0) | 1042 (9.7) | 368 (12.5) | 89 (6.9) |
| Other | 2869 (19.1) | 2238 (20.8) | 455 (15.4) | 176 (13.6) |
| Grade |  |  |  |  |
| Ⅰ | 937 (6.2) | 636 (5.9) | 214 (7.3) | 87 (6.7) |
| Ⅱ | 5094 (33.9) | 3582 (33.2) | 1095 (37.1) | 417 (32.3) |
| Ⅲ/Ⅳ | 6110 (40.7) | 4053 (37.6) | 1398 (47.4) | 659 (51.1) |
| Unknown | 2871 (19.1) | 2503 (23.2) | 241 (8.2) | 127 (9.8) |
| AJCC T category |  |  |  |  |
| 0 | 240 (1.6) | 237 (2.2) | 2 (0.1) | 1 (0.1) |
| 1 | 1802 (12.0) | 1206 (11.2) | 480 (16.3) | 116 (9.0) |
| 2 | 4390 (29.2) | 2776 (25.8) | 1257 (42.6) | 357 (27.7) |
| 3 | 2305 (15.4) | 1534 (14.2) | 532 (18.0) | 239 (18.5) |
| 4 | 4405 (29.3) | 3319 (30.8) | 576 (19.5) | 510 (39.5) |
| X | 1870 (12.5) | 1702 (15.8) | 101 (3.4) | 67 (5.2) |
| AJCC N category |  |  |  |  |
| 0 | 3199 (21.3) | 2369 (22.0) | 628 (21.3) | 202 (15.7) |
| 1 | 6764 (45.1) | 5242 (48.7) | 925 (31.4) | 597 (46.3) |
| 2 | 1649 (11.0) | 816 (7.6) | 620 (21.0) | 213 (16.5) |
| 3 | 2278 (15.2) | 1378 (12.8) | 665 (22.6) | 235 (18.2) |
| X | 1122 (7.5) | 969 (9.0) | 110 (3.7) | 43 (3.3) |

Table 1. (continued)

| Variables | All patients  (N = 15012) | Systemic therapy without primary surgery  (N = 10774) | Systemic therapy after primary surgery  (N = 2948) | Systemic therapy before primary surgery  (N = 1290 ) |
| --- | --- | --- | --- | --- |
| Molecular subtype |  |  |  |  |
| HR+/HER2– | 7862 (52.4) | 5428 (50.4) | 1828 (62.0) | 606 (47.0) |
| HR+/HER2+ | 2534 (16.9) | 1908 (17.7) | 412 (14.0) | 214 (16.6) |
| HR-/HER2+ | 1422 (9.5) | 1067 (9.9) | 214 (7.3) | 141 (10.9) |
| HR-/HER2– | 2037 (13.6) | 1448 (13.4) | 334 (11.3) | 255 (19.8) |
| Unknown | 1157 (7.7) | 923 (8.6) | 160 (5.4) | 74 (5.7) |
| Bone involvement |  |  |  |  |
| No | 3856 (25.7) | 2599 (24.1) | 812 (27.5) | 445 (34.5) |
| Yes | 11156 (74.3) | 8175 (75.9) | 2136 (72.5) | 845 (65.5) |
| Lung involvement |  |  |  |  |
| No | 9791 (65.2) | 6796 (63.1) | 2125 (72.1) | 870 (67.4) |
| Yes | 5221 (34.8) | 3978 (36.9) | 823 (27.9) | 420 (32.6) |
| Liver involvement |  |  |  |  |
| No | 10438 (69.5) | 7160 (66.5) | 2270 (77.0) | 1008 (78.1) |
| Yes | 4574 (30.5) | 3614 (33.5) | 678 (23.0) | 282 (21.9) |
| Brain involvement |  |  |  |  |
| No | 13746 (91.6) | 9696 (90.0) | 2814 (95.5) | 1236 (95.8) |
| Yes | 1266 (8.4) | 1078 (10.0) | 134 (4.5) | 54 (4.2) |
| Site of metastasis |  |  |  |  |
| Bone only | 6214 (41.4) | 4056 (37.6) | 1537 (52.1) | 621 (48.1) |
| Viscera only | 3415 (22.7) | 2249 (20.9) | 755 (25.6) | 411 (31.9) |
| Bone + viscera | 4117 (27.4) | 3391 (31.5) | 522 (17.7) | 204 (15.8) |
| Brain involvement | 1266 (8.4) | 1078 (10.0) | 134 (4.5) | 54 (4.2) |
| Number of sites of metastasis |  |  |  |  |
| 1 | 9447 (62.9) | 6153 (57.1) | 2264 (76.8) | 1030 (79.8) |
| 2 | 4121 (27.5) | 3355 (31.1) | 555 (18.8) | 211 (16.4) |
| ≥ 3 | 1444 (9.6) | 1266 (11.8) | 129 (4.4) | 49 (3.8) |
| Vital status |  |  |  |  |
| Alive | 6761 (45.0) | 4903 (45.5) | 1250 (42.4) | 608 (47.1) |
| Dead of breast cancer | 7319 (48.8) | 5228 (48.5) | 1478 (50.1) | 613 (47.5) |
| Dead of other cause | 932 (6.2) | 643 (6.0) | 220 (7.5) | 69 (5.3) |
| Radiation therapy |  |  |  |  |
| None/unknown | 10246 (68.3) | 7673 (71.2) | 1896 (64.3) | 677 (52.5) |
| Yes | 4766 (31.7) | 3101 (28.8) | 1052 (35.7) | 613 (47.5) |
| Non-primary surgical procedure to distant site | | | | |
| No | 14409 (96.0) | 10358 (96.1) | 2811 (95.4) | 1240 (96.1) |
| Yes | 475 (3.2) | 66 (0.6) | 40 (1.4) | 22 (1.7) |
| Unknown | 128 (0.9) | 350 (3.2) | 97 (3.3) | 28 (2.2) |

Table 1. (continued)

| Variables | All patients  (N = 15012) | Systemic therapy without primary surgery  (N = 10774) | Systemic therapy after primary surgery  (N = 2948) | Systemic therapy before primary surgery  (N = 1290 ) |
| --- | --- | --- | --- | --- |
| Surgical procedure |  |  |  |  |
| Partial mastectomy | |  | 1111 (37.7) | 249 (19.3) |
| Subcutaneous/simple mastectomy | |  | 707 (24.0) | 416 (32.2) |
| Modified radical mastectomy | |  | 1054 (35.8) | 574 (44.5) |
| (Extended) Radical mastectomy | |  | 34 (1.2) | 19 (1.5) |
| Unknown | |  | 42 (1.4) | 32 (2.5) |

Abbreviations: IQR, interquartile range; IDC, invasive ductal carcinoma; ILC, invasive lobular carcinoma; HR, hormone receptor; HER2, human epidermal growth factor receptor 2

The *P* value for comparison of categorical variables across groups is 0.03 for marital status, 0.001 for vital status, and < 0.001 for other variables.

Table 2. A logistic model predicting the type of surgical procedure (N = 4238)

| Surgical Procedure | Treatment modality | OR (95% CI) | *P-value* |
| --- | --- | --- | --- |
| Subcutaneous/simple mastectomy | Systemic therapy after surgery | 1 [Reference] |  |
|  | Systemic therapy before surgery | 2.32 (1.97-2.74) | < 0.001 |
| Modified radical mastectomy | Systemic therapy after surgery | 1 [Reference] |  |
|  | Systemic therapy before surgery | 2.23 (1.90-2.63) | < 0.001 |
| (Extended) radical mastectomy | Systemic therapy after surgery | 1 [Reference] |  |
|  | Systemic therapy before surgery | 2.27 (2.22-2.31) | < 0.001 |

Only patients who underwent combined treatment with systemic therapy and surgery of the primary site were included in this model. The logistic regression model was adjusted for year of diagnosis, age, grade, T category, N category, and number of sites of metastasis. The variables above were selected using the stepwise AIC method in both directions. The reference category of surgical procedure is partial mastectomy.

Abbreviations: OR, odds ratio; CI, confidence interval

Table 3. Univariate and multivariate analysis of BCSD: a competing risk regression model

| Variables | Univariate Analysis | | Multivariate Analysis | |
| --- | --- | --- | --- | --- |
|  | SHR (95% CI) | *P-value* | SHR (95% CI) | *P-value* |
| Treatment modality |  |  |  |  |
| Systemic therapy without primary surgery | 1 [Reference] | NA | 1 [Reference] | NA |
| Systemic therapy after primary surgery | 0.73 (0.69-0.77) | < 0.001 | 0.74 (0.69-0.79) | < 0.001 |
| Systemic therapy before primary surgery | 0.67 (0.62-0.73) | < 0.001 | 0.62 (0.56-0.67) | < 0.001 |
| Treatment modality (reference category changed) | | | | |
| Systemic therapy without primary surgery | - | - | - | - |
| Systemic therapy after primary surgery | 1 [Reference] | NA | 1 [Reference] | NA |
| Systemic therapy before primary surgery | 0.92 (0.84-1.01) | 0.07 | 0.83 (0.76-0.92) | < 0.001 |
| Year of diagnosis |  |  |  |  |
| As a continuous variable | 0.95 (0.94-0.96) | < 0.001 | 0.94 (0.93-0.95) | < 0.001 |
| Age, y |  |  |  |  |
| [18, 40) | 1 [Reference] | NA | 1 [Reference] | NA |
| [40, 60) | 1.14 (1.04-1.24) | 0.003 | 1.13 (1.04-1.23) | 0.006 |
| [60, 100) | 1.25 (1.15-1.36) | < 0.001 | 1.28 (1.18-1.40) | < 0.001 |
| Marital status |  |  |  |  |
| Unmarried | 1 [Reference] | NA | 1 [Reference] | NA |
| Married | 0.85 (0.82-0.89) | < 0.001 | 0.91 (0.86-0.95) | < 0.001 |
| Unknown | 0.95 (0.85-1.06) | 0.36 | 0.96 (0.85-1.07) | 0.46 |
| Race |  |  |  |  |
| White | 1 [Reference] | NA | 1 [Reference] | NA |
| Black | 1.35 (1.27-1.43) | < 0.001 | 1.20 (1.12-1.28) | < 0.001 |
| Other | 0.94 (0.86-1.03) | 0.16 | 0.95 (0.87-1.04) | 0.27 |
| Unknown | 0.50 (0.29-0.86) | 0.01 | 0.49 (0.28-0.85) | 0.01 |
| Sex |  |  |  |  |
| Female | 1 [Reference] | NA | - | - |
| Male | 0.98 (0.79-1.2) | 0.83 | - | - |
| Histologic type |  |  |  |  |
| IDC | 1 [Reference] | NA | 1 [Reference] | NA |
| ILC | 0.99 (0.92-1.06) | 0.70 | 1.25 (1.15-1.36) | < 0.001 |
| Other | 1.15 (1.09-1.22) | < 0.001 | 1.08 (1.01-1.16) | 0.02 |
| Grade |  |  |  |  |
| Ⅰ | 1 [Reference] | NA | 1 [Reference] | NA |
| Ⅱ | 1.22 (1.10-1.35) | < 0.001 | 1.22 (1.10-1.36) | < 0.001 |
| Ⅲ/Ⅳ | 1.81 (1.63-2.00) | < 0.001 | 1.63 (1.47-1.82) | < 0.001 |
| Unknown | 1.66 (1.49-1.85) | < 0.001 | 1.36 (1.22-1.53) | < 0.001 |

Table 3. (continued)

| Variables | Univariate Analysis | | Multivariate Analysis | |
| --- | --- | --- | --- | --- |
|  | SHR (95% CI) | *P-value* | SHR (95% CI) | *P-value* |
| AJCC T category |  |  |  |  |
| 0/1 | 1 [Reference] | NA | 1 [Reference] | NA |
| 2 | 1.08 (1.00-1.17) | 0.05 | 1.12 (1.03-1.22) | 0.008 |
| 3 | 1.36 (1.24-1.48) | < 0.001 | 1.26 (1.15-1.38) | < 0.001 |
| 4 | 1.54 (1.42-1.66) | < 0.001 | 1.32 (1.21-1.44) | < 0.001 |
| X | 1.43 (1.31-1.57) | < 0.001 | 1.23 (1.11-1.36) | < 0.001 |
| AJCC N category |  |  |  |  |
| 0 | 1 [Reference] | NA | 1 [Reference] | NA |
| 1 | 1.03 (0.97-1.09) | 0.34 | 0.96 (0.90-1.03) | 0.27 |
| 2 | 1.03 (0.95-1.12) | 0.42 | 1.04 (0.95-1.13) | 0.42 |
| 3 | 1.18 (1.09-1.27) | < 0.001 | 1.10 (1.01-1.19) | 0.03 |
| X | 1.24 (1.12-1.37) | < 0.001 | 0.99 (0.88-1.11) | 0.83 |
| Molecular subtype |  |  |  |  |
| HR+/HER2– | 1 [Reference] | NA | 1 [Reference] | NA |
| HR+/HER2+ | 0.81 (0.76-0.87) | < 0.001 | 0.70 (0.65-0.75) | < 0.001 |
| HR-/HER2+ | 1.02 (0.94-1.11) | 0.60 | 0.86 (0.78-0.94) | < 0.001 |
| HR-/HER2– | 2.52 (2.36-2.70) | < 0.001 | 2.15 (1.99-2.32) | < 0.001 |
| Unknown | 1.27 (1.17-1.37) | < 0.001 | 1.06 (0.97-1.16) | 0.20 |
| Site of metastasis |  |  |  |  |
| Bone only | 1 [Reference] | NA | 1 [Reference] | NA |
| Viscera only | 1.23 (1.16-1.31) | < 0.001 | 1.06 (0.99-1.14) | 0.10 |
| Bone + viscera | 1.62 (1.54-1.71) | < 0.001 | 1.09 (0.94-1.25) | 0.26 |
| Brain involvement | 2.26 (2.08-2.47) | < 0.001 | 1.38 (1.18-1.60) | < 0.001 |
| Number of sites of metastasis |  |  |  |  |
| 1 | 1 [Reference] | NA | 1 [Reference] | NA |
| 2 | 1.50 (1.43-1.58) | < 0.001 | 1.33 (1.17-1.52) | < 0.001 |
| ≥ 3 | 2.06 (1.91-2.22) | < 0.001 | 1.66 (1.43-1.94) | < 0.001 |
| Radiation therapy |  |  |  |  |
| None/unknown | 1 [Reference] | NA | 1 [Reference] | NA |
| Yes | 1.07 (1.02-1.12) | 0.005 | 1.06 (1.00-1.12) | 0.04 |
| Non-primary surgical procedure to distant site | | | | |
| No | 1 [Reference] | NA | 1 [Reference] | NA |
| Yes | 0.83 (0.73-0.96) | 0.009 | 0.80 (0.69-0.93) | 0.003 |
| Unknown | 0.79 (0.62-1.00) | 0.05 | 0.92 (0.73-1.17) | 0.52 |

Abbreviations: SHR, subdistribution hazard ratio; CI, confidence interval; NA, not applicable; IDC, invasive ductal carcinoma; ILC, invasive lobular carcinoma

Table 4. Multivariate analysis of BCSD according to the metastatic site in patients with single-organ involvement

|  | Bone-only metastasis  (N = 6214) | | Lung-only metastasis  (N = 1725) | | Liver-only metastasis  (N = 1300) | | Brain-only metastasis  (N = 208) | |
| --- | --- | --- | --- | --- | --- | --- | --- | --- |
|  | SHR (95% CI) | *P-value* | SHR (95% CI) | *P-value* | SHR (95% CI) | *P-value* | SHR (95% CI) | *P-value* |
| Treatment modality in Model 1 |  |  |  |  |  |  |  |  |
| Systemic therapy without primary surgery | 1 [Reference] | NA | 1 [Reference] | NA | 1 [Reference] | NA | 1 [Reference] | NA |
| Systemic therapy after primary surgery | 0.66 (0.60-0.74) | < 0.001 | 0.81 (0.68-0.97) | 0.02 | 0.79 (0.64-0.97) | 0.03 | 0.89 (0.57-1.39) | 0.61 |
| Systemic therapy before primary surgery | 0.61 (0.53-0.69) | < 0.001 | 0.58 (0.46-0.72) | < 0.001 | 0.62 (0.47-0.83) | < 0.001 | 0.64 (0.33-1.26) | 0.20 |
| Treatment modality in Model 2 |  |  |  |  |  |  |  |  |
| Systemic therapy without primary surgery | - | - | - | - | - | - | - | - |
| Systemic therapy after primary surgery | 1 [Reference] | NA | 1 [Reference] | NA | 1 [Reference] | NA | 1 [Reference] | NA |
| Systemic therapy before primary surgery | 0.92 (0.80-1.06) | 0.23 | 0.71 (0.56-0.90) | 0.005 | 0.79 (0.58-1.08) | 0.13 | 0.72 (0.37-1.41) | 0.34 |
| Non-primary surgical procedure to distant site |  |  |  |  |  |  |  |  |
| No | 1 [Reference] | NA | 1 [Reference] | NA | 1 [Reference] | NA | 1 [Reference] | NA |
| Yes | 0.96 (0.75-1.22) | 0.72 | 0.61 (0.36-1.03) | 0.07 | 0.78 (0.46-1.33) | 0.36 | 0.90 (0.55-1.46) | 0.67 |
| Unknown | 1.34 (0.95-1.87) | 0.09 | 0.46 (0.21-1.00) | 0.05 | 0.55 (0.18-1.69) | 0.30 | 0.94 (0.34-2.60) | 0.90 |

The reference category of treatment modality on primary site varied within different models (“systemic therapy without primary surgery” for Model 1 and “systemic therapy after primary surgery” for Model 2). The competing risk model was adjusted for age, race, marital status, year of diagnosis, histologic type, grade, molecular subtype, AJCC T category, AJCC N category, receipt of radiotherapy, and non-primary surgical procedure to distant site.

Abbreviations: SHR, subdistribution hazard ratio; CI, confidence interval; NA, not applicable
