# Supplementary figures and images for: Exploring the Value of Additional Primary Tumour Excision Combined with Systemic Therapy Administered in Different Sequences for Patients with de Novo Metastatic Breast Cancer
Source: Breast J. 2022 Aug 25;2022:5049445. doi: 10.1155/2022/5049445 (PMC9436631; doi:10.1155/2022/5049445)

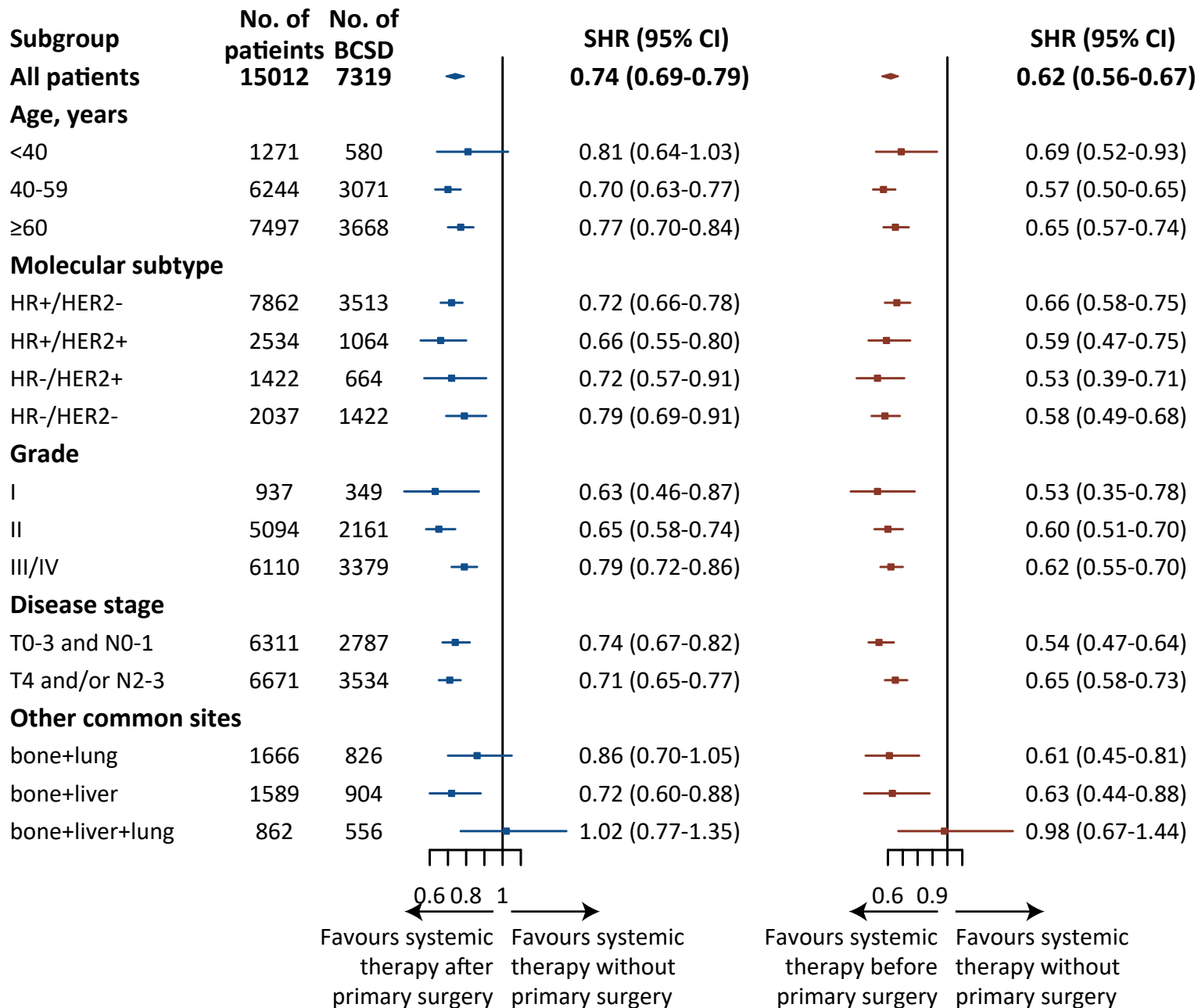

Supplement: Supplementary Materials — Supplementary Table 1: Multivariate analysis of all-cause death: a Cox proportional hazards model. Supplementary Table 2: Baseline characteristics of patients in the group of systemic therapy without primary surgery and the group of systemic therapy after primary surgery after propensity score matching. Supplementary Table 3: Baseline characteristics of patients in the group of systemic therapy without primary surgery and the group of systemic therapy before primary surgery after propensity score matching. Supplementary Table 4: Multivariate analysis of BCSS and OS after 1 : 1 matching the cases from the group of systemic therapy without primary surgery and the group of systemic therapy after primary surgery. Supplementary Table 5: Multivariate analysis of BCSS and OS after 1 : 1 matching the cases from the group of systemic therapy without primary surgery and the group of systemic therapy before primary surgery. Supplementary Table 6: Multivariate analysis of all-cause death according to the metastatic site in patients with single-organ involvement. Supplementary Figure 1: Kaplan–Meier curves of OS in patients with single-organ disease involving the bone (A), lung (B), liver (C), and brain (D). Supplementary Figure 2: Forest plot of subgroup analysis on BCSD, adjusted subdistribution hazard ratios. Supplementary Figure 3: Forest plot of subgroup analysis on all-cause death, adjusted hazard ratios, 15. [file 5049445.f1.zip › 5049445.f1/Supplementary Figure 2. Forest plot of subgroup analysis on BCSD, adjusted subdistribution hazard ratios (2).pdf]

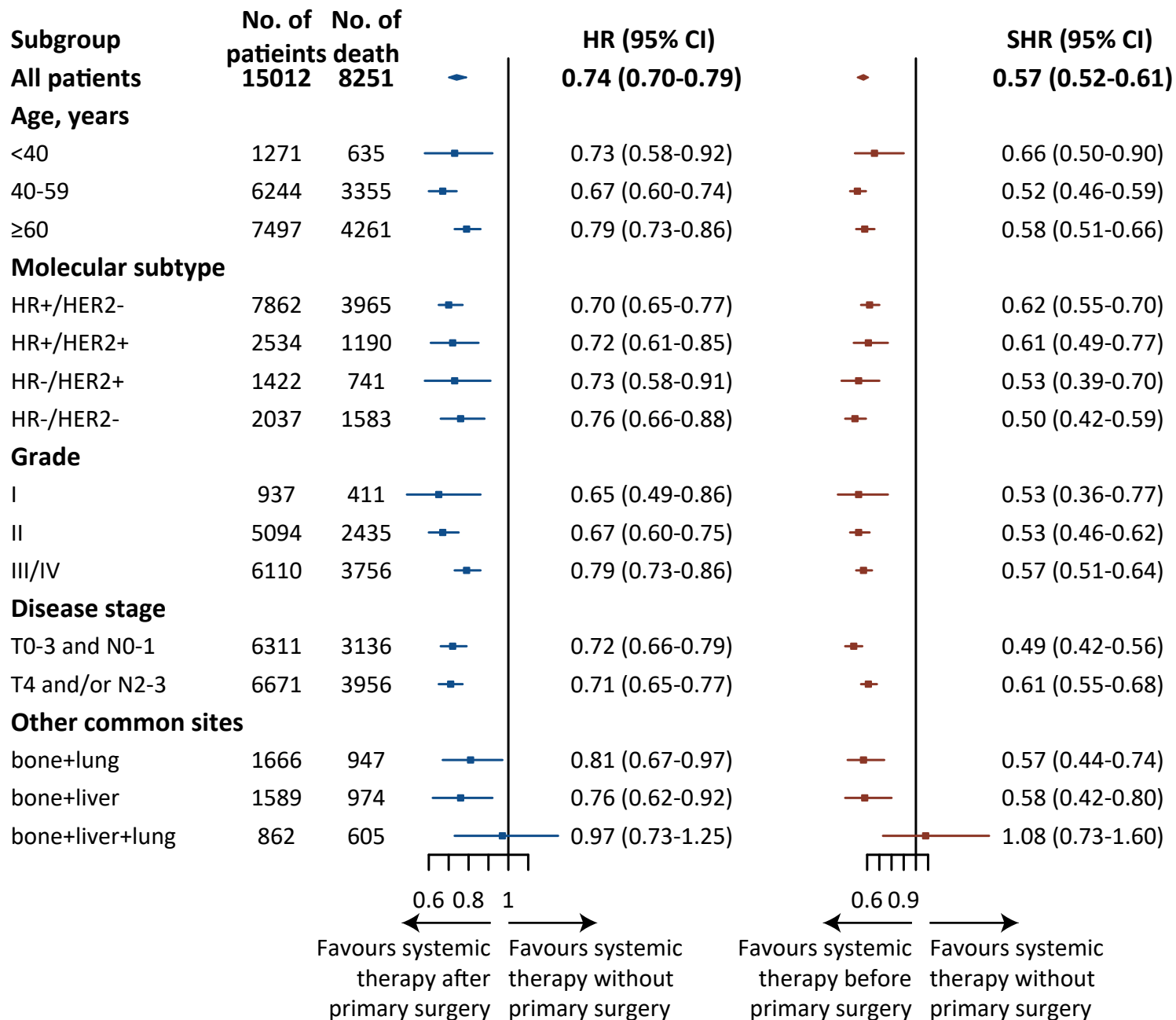

Supplement: Supplementary Materials — Supplementary Table 1: Multivariate analysis of all-cause death: a Cox proportional hazards model. Supplementary Table 2: Baseline characteristics of patients in the group of systemic therapy without primary surgery and the group of systemic therapy after primary surgery after propensity score matching. Supplementary Table 3: Baseline characteristics of patients in the group of systemic therapy without primary surgery and the group of systemic therapy before primary surgery after propensity score matching. Supplementary Table 4: Multivariate analysis of BCSS and OS after 1 : 1 matching the cases from the group of systemic therapy without primary surgery and the group of systemic therapy after primary surgery. Supplementary Table 5: Multivariate analysis of BCSS and OS after 1 : 1 matching the cases from the group of systemic therapy without primary surgery and the group of systemic therapy before primary surgery. Supplementary Table 6: Multivariate analysis of all-cause death according to the metastatic site in patients with single-organ involvement. Supplementary Figure 1: Kaplan–Meier curves of OS in patients with single-organ disease involving the bone (A), lung (B), liver (C), and brain (D). Supplementary Figure 2: Forest plot of subgroup analysis on BCSD, adjusted subdistribution hazard ratios. Supplementary Figure 3: Forest plot of subgroup analysis on all-cause death, adjusted hazard ratios, 15. [file 5049445.f1.zip › 5049445.f1/Supplementary Figure 3. Forest plot of subgroup analysis on all-cause death, adjusted hazard ratios (2).pdf]
